# Evaluating Alternative Ramucirumab Doses as a Single Agent or with Paclitaxel in Second-Line Treatment of Locally Advanced or Metastatic Gastric/Gastroesophageal Junction Adenocarcinoma: Results from Two Randomized, Open-Label, Phase II Studies

**DOI:** 10.3390/cancers14051168

**Published:** 2022-02-24

**Authors:** Manish A. Shah, Anghel Adrian Udrea, Igor Bondarenko, Was Mansoor, Raquel Guardeño Sánchez, Tomasz Sarosiek, Silvia Bozzarelli, Michael Schenker, Carlos Gomez-Martin, Carys Morgan, Mustafa Özgüroğlu, Joanna Pikiel, Haralabos P. Kalofonos, Elzbieta Wojcik, Tomas Buchler, Daniel Swinson, Irfan Cicin, Mano Joseph, Ihor Vynnychenko, Alexander Valerievich Luft, Peter C. Enzinger, Tomas Salek, Christos Papandreou, Christophe Tournigand, Evaristo Maiello, Ran Wei, David Ferry, Ling Gao, Joana M. Oliveira, Jaffer A. Ajani

**Affiliations:** 1 Department of Medicine, Division of Hematology and Medical Oncology, Weill Cornell Medical College, New York, NY 10021, USA; 2 Cancer Center, Medisprof SRL, 400641 Cluj-Napoca, Romania; adrianudrea@medisprof.ro; 3 Department of Oncology, Dnipropetrovsk Medical Academy, 49044 Dnipropetrovsk, Ukraine; igor.bondarenko@rdp-ukraine.com; 4 Department of Medical Oncology, The Christie NHS Foundation Trust, Manchester M20 4BX, UK; was.mansoor@christie.nhs.uk; 5 Department of Medical Oncology, Catalan Institute of Oncology (ICO) Girona Hospital Dr Josep Trueta, 17007 Girona, Spain; rguardeno@iconcologia.net; 6 Department of Clinical Oncology and Oncological Surgery, LUXMED Onkologia, 04125 Warszawa, Poland; tomasz.sarosiek@luxmed.pl; 7 Medical Oncology and Hematology Unit, Humanitas Cancer Center, IRCCS Humanitas Research Hospital, 20089 Milan, Italy; silvia.bozzarelli@cancercenter.humanitas.it; 8 Centrul de Oncologie Sf. Nectarie SRL, 200542 Craiova, Romania; michael.schenker@umfcv.ro; 9 Department of Medical Oncology, University of Medicine and Pharmacy Craiova, 200342 Craiova, Romania; 10 Medical Oncology Department, Hospital Universitario 12 de Octubre, 28041 Madrid, Spain; cgomezm@seom.org; 11 Department of Clinical Oncology, Velindre Cancer Centre, Cardiff CF14 2TL, UK; carys.morgan6@wales.nhs.uk; 12 Medical Oncology, Istanbul University, Cerrahpaşa, Fatih, Istanbul 34098, Turkey; mozgur@iuc.edu; 13 Department of Oncology, Copernicus Podmiot Leczniczy, 80-803 Gdańsk, Poland; joanna.pikiel@post.pl; 14 Department of Oncology, University General Hospital of Patras Rion, 26504 Patras, Greece; kalofonos@upatras.gr; 15 NZOZ Centrum Medyczne HCP, 62-030 Luboń, Poland; onkologia.hcp@onet.eu; 16 Department of Oncology, First Faculty of Medicine, Charles University and Thomayer University Hospital, 14059 Prague, Czech Republic; tomas.buchler@ftn.cz; 17 Institute of Oncology, St James’s University Hospital, Leeds LS9 7TF, UK; daniel.swinson@nhs.net; 18 Medical Oncology, Trakya University, Edirne 22030, Turkey; irfancicin@trakya.edu.tr; 19 Deanesly Centre, New Cross Hospital, Wolverhamptom WV10 0QP, UK; mano.joseph@nhs.net; 20 Sumy Regional Oncology Center, Sumy State University, 40000 Sumy, Ukraine; i.vynnychenko@med.sumdu.edu.ua; 21 Department of Oncology No 1 (Thoracic Surgery), Leningrad Regional Clinical Hospital, 194291 St. Petersburg, Russia; alexander_luft@mail.ru; 22 Medical Oncology, Dana-Farber Cancer Institute, Boston, MA 02215, USA; peter_enzinger@dfci.harvard.edu; 23 Department of Clinical Oncology, Narodny Onkologicky Ustav, 83310 Bratislava, Slovakia; tomas.salek@nou.sk; 24 Department of Medical Oncology, Faculty of Medicine, University of Thessaly, Biopolis, 41223 Larissa, Greece; cpapandr@otenet.gr; 25 Department of Medical Oncology, Henri Mondor et Albert Chenevier Teaching Hospital, Assistance Publique-Hôpitaux de Paris, University of Paris-Est Creteil, 94000 Créteil, France; christophe.tournigand@aphp.fr; 26 Oncology Unit, Foundation Casa Sollievo della Sofferenza IRCCS, Viale Cappuccini 1, 71013 San Giovanni Rotondo, Italy; e.maiello@libero.it; 27 Eli Lilly and Company, Indianapolis, IN 46225, USA; wei_ran@lilly.com; 28 Eli Lilly and Company, New York, NY 10016, USA; ferry_david@lilly.com (D.F.); linggao418@gmail.com (L.G.); oliveira_joana@lilly.com (J.M.O.); 29 Department of Gastrointestinal Medical Oncology, University of Texas MD Anderson Cancer Center, Houston, TX 77030, USA; jajani@mdanderson.org

**Keywords:** angiogenesis, gastric adenocarcinoma, ramucirumab, vascular endothelial growth factor receptor

## Abstract

**Simple Summary:**

Ramucirumab is indicated at a dosage of 8 mg/kg every 2 weeks as monotherapy or in combination with paclitaxel for second-line advanced/metastatic gastric/gastroesophageal junction (GEJ) adenocarcinoma. A post hoc analysis of the phase III trials REGARD and RAINBOW suggested a positive correlation between ramucirumab exposure and efficacy. Studies JVDB and JVCZ explored different ramucirumab dosing regimens as monotherapy and in combination with paclitaxel, respectively. Here we report results from these studies, in which JVDB evaluated the pharmacokinetics and safety of the currently registered dosing regimen for ramucirumab monotherapy and three exploratory dosing regimens, and JVCZ evaluated the efficacy and safety of a higher dosing regimen of ramucirumab in combination with paclitaxel in second-line gastric/GEJ adenocarcinoma. Overall, the safety profiles were similar between the registered dose and the exploratory dosing regimens. However, a lack of a dose/exposure-response relationship supports the standard dose of ramucirumab as second-line treatment for patients with advanced/metastatic gastric/GEJ adenocarcinoma.

**Abstract:**

Studies JVDB and JVCZ examined alternative ramucirumab dosing regimens as monotherapy or combined with paclitaxel, respectively, in patients with advanced/metastatic gastric/gastroesophageal junction (GEJ) adenocarcinoma. For JVDB, randomized patients (*N* = 164) received ramucirumab monotherapy at four doses: 8 mg/kg every 2 weeks (Q2W) (registered dose), 12 mg/kg Q2W, 6 mg/kg weekly (QW), or 8 mg/kg on days 1 and 8 (D1D8) every 3 weeks (Q3W). The primary objectives were the safety and pharmacokinetics of ramucirumab monotherapy. For JVCZ, randomized patients (*N* = 245) received paclitaxel (80 mg/m^2^-D1D8D15) plus ramucirumab (8 mg/kg- or 12 mg/kg-Q2W). The primary objective was progression-free survival (PFS) of 12 mg/kg-Q2W arm versus placebo from RAINBOW using meta-analysis. Relative to the registered dose, exploratory dosing regimens (EDRs) led to higher ramucirumab serum concentrations in both studies. EDR safety profiles were consistent with previous studies. In JVDB, serious adverse events occurred more frequently in the 8 mg/kg-D1D8-Q3W arm versus the registered dose; 6 mg/kg-QW EDR had a higher incidence of bleeding/hemorrhage. In JVCZ, PFS was improved with the 12 mg/kg plus paclitaxel combination versus placebo in RAINBOW; however, no significant PFS improvement was observed between the 12 mg/kg and 8 mg/kg arms. The lack of a dose/exposure-response relationship in these studies supports the standard dose of ramucirumab 8 mg/kg-Q2W as monotherapy or in combination with paclitaxel as second-line treatment for advanced/metastatic gastric/GEJ adenocarcinoma.

## 1. Introduction

Ramucirumab is a human IgG1 monoclonal antibody that binds to the extracellular domain of the vascular endothelial growth factor receptor-2 (VEGFR-2), blocking binding of VEGF-A, VEGF-C, and VEGF-D [1] and inhibiting tumor angiogenesis [2]. Ramucirumab has shown antitumor activity in phase III trials and has received approval for the treatment of several tumor types, including gastric or gastroesophageal junction (GEJ) adenocarcinomas [3,4,5,6,7]. REGARD [4] and RAINBOW [5] were the pivotal randomized phase III trials that established the safety and efficacy of ramucirumab 8 mg/kg every 2 weeks (Q2W) alone [4] or in combination with paclitaxel [5] in patients with previously treated gastric/GEJ adenocarcinoma.

Subsequent exploratory exposure-response analyses of REGARD and RAINBOW data, and a case-control analysis of the latter, suggested higher ramucirumab exposure was associated with longer overall survival (OS) and progression-free survival (PFS) [8,9]. In the exposure-response analysis of RAINBOW, an increased risk of grade ≥3 hypertension, leukopenia, and neutropenia, but not febrile neutropenia, was correlated with increased ramucirumab exposure [8]; however, these toxicities were manageable in the original phase III trials [4,5]. These exposure-response findings, and the fact that the ramucirumab maximum tolerated dose (MTD) is 13 mg/kg/week [2], suggested there may be an opportunity to further improve efficacy while maintaining an acceptable safety profile. Thus, 2 phase II post-marketing commitment studies were conducted: JVDB and JVCZ. Here, we describe the results from these trials, where JVDB evaluated the pharmacokinetics (PK) and safety of the currently registered dosing regimen for ramucirumab monotherapy and three exploratory dosing regimens (EDRs), and JVCZ evaluated the efficacy and safety of a higher dosing regimen of ramucirumab in combination with paclitaxel in second-line gastric/GEJ adenocarcinoma.

## 2. Materials and Methods

### 2.1. Study Design

Studies JVDB (NCT02443883) and JVCZ (NCT02514551) were multinational, open label, randomized, phase II clinical trials that compared alternative ramucirumab doses as monotherapy (JVDB) or combined with paclitaxel (JVCZ) in patients with metastatic or locally advanced gastric/GEJ adenocarcinoma. Eligible patients were aged ≥18 with measurable disease according to Response Evaluation Criteria in Solid Tumors version 1.1 (RECISTv1.1) [10], and had an Eastern Cooperative Oncology Group performance status (ECOG PS) of 0 or 1. Key exclusion criteria for both trials included having received >1 line of therapy, prior treatment with taxanes (for JVCZ only), and antiangiogenic agents. Full eligibility criteria are provided in the Online Supplementary Methods. All patients provided written informed consent before participation. The protocol was approved by the ethics committee for all participating centers. The study adhered to the Declaration of Helsinki and the International Conference on Harmonization Guidelines for Good Clinical Practice, and applicable local regulations.

### 2.2. Study Treatments

In JVDB, patients were randomized 1:1:1:1 to receive ramucirumab intravenously in 1 of 4 dosing regimens as shown in Figure S1A: Arm 1, 8 mg/kg (current registered dose) on days 1 and 15 of a 28-day cycle (Q2W) [1]; Arm 2, 12 mg/kg (Q2W); Arm 3, 6 mg/kg weekly (on days 1, 8, 15, and 22 of a 28-day cycle [QW]); Arm 4, 8 mg/kg on days 1 and 8 of a 21-day cycle (D1D8-Q3W). Stratification factors were body weight (<60 kg vs. ≥60 kg) and ECOG PS (0 vs. 1).

In JVCZ, patients were randomized 1:1 after ECOG PS (0 vs. 1) stratification to 1 of the 2 dosing regimens: either 8 mg/kg Q2W (the currently registered dose) or 12 mg/kg Q2W ramucirumab, both in combination with paclitaxel at 80 mg/m^2^ on days 1, 8, and 15 of a 28-day cycle (Figure S1B).

Based on PK simulations, the ramucirumab EDRs for both studies were expected to produce higher exposure and potentially better clinical activity outcomes relative to the 8 mg/kg-Q2W dosing regimen.

### 2.3. Study Assessments

Tumor responses were assessed radiographically according to RECISTv1.1 [11] during study treatment, every 6 weeks (±7 days) for the first 6 months in study JVDB, and every 9 weeks (±7 days) thereafter. In JVCZ, imaging was performed every 8 weeks (±7 days). Treatment continued until disease progression, unacceptable toxicity, or discontinuation for any other reason. Adverse events (AEs) were evaluated throughout the study and for 30 days after treatment discontinuation according to the Common Terminology Criteria for Adverse Events, Version 4.0, and were judged by the investigator as related or unrelated to study treatment. PK and immunogenicity sampling was carried out according to Table S1. Ramucirumab serum concentrations were analyzed using a validated Enzyme-Linked Immunosorbent Assay (ELISA, Intertek Pharmaceutical Services, San Diego, CA, USA). Immunogenicity testing was performed using a validated assay (BioAgilytix Inc., Durham, NC, USA).

### 2.4. Outcomes

For JVDB, the primary objective was to evaluate the PK and safety of various dosing regimens of ramucirumab monotherapy. Secondary objectives were PFS rate at the first 6-week tumor assessment (6-week PFS rate) as determined by the investigator per RECISTv1.1, and immunogenicity for ramucirumab. Exploratory objectives were PFS, ORR, and OS.

For JVCZ, the primary objective was to compare PFS between ramucirumab 12 mg/kg plus paclitaxel and placebo plus paclitaxel using RAINBOW data (PFS Analysis 1) through inter-trial analysis. The key assumption was that the study populations in JVCZ and RAINBOW were similar with respect to predictive and prognostic factors. Secondary objectives were PFS between ramucirumab 12 versus 8 mg/kg (PFS Analysis 2), PK, safety and tolerability, objective response rate (ORR), disease control rate (DCR), and immunogenicity. The trial was not powered for OS, which was an exploratory objective. Efficacy measures are defined in Online Supplementary Methods.

### 2.5. Statistical Analyses

Safety analyses were based on all enrolled patients who received at least 1 dose of ramucirumab (JVDB), or a partial dose of ramucirumab or paclitaxel (JVCZ). Efficacy analyses were based on the intention-to-treat population, defined as all patients randomized to study treatment.

For JVDB, a sample size of 160 patients (40 per arm) was needed to provide an adequate estimate of the PFS rate at 6 weeks based on an assumed 6-week PFS rate of about 60% for Arm 1 (based on REGARD study) and 80% for the exploratory doses, with a 50% chance to detect a statistical difference at α = 0.05.

For JVCZ, an assumed 2-month increase in median PFS between ramucirumab 12 versus 8 mg/kg (PFS Analysis 2) with an estimated hazard ratio (HR) = 0.667 (HR2) was used to estimate the sample size of 191 PFS events observed in JVCZ study. In the primary efficacy analysis (PFS Analysis 1), comparing the PFS between ramucirumab 12 mg/kg and the placebo arm in RAINBOW, HR was calculated by HR1*HR2 using meta-analysis. HR1 = 0.635 was the observed HR for PFS from RAINBOW and HR2 (ramucirumab 12 vs. 8 mg/kg) was estimated using the Cox proportional hazard model based on the JVCZ data only. Only 64 PFS events were needed to achieve a statistical power of 90% for PFS Analysis 1 and thus the sample size of 191 events as determined by PFS Analysis 2 was also sufficient for the meta-analysis in PFS Analysis 1.

In both trials, time-to-event variables for both PFS and OS were estimated using Kaplan–Meier methods with 95% confidence intervals (CIs). All tests of treatment effects were conducted using the log-rank test at a 2-sided alpha-level of 0.05. A Cox proportional hazard model was used to estimate the HR between the treatment arms and the corresponding CIs and Wald *p*-values. The proportions of patients achieving objective response and disease control between treatment arms were compared using the Cochran–Mantel–Haenzel test. Statistical analyses were performed using SAS software (SAS, Version 9.1.2 or higher, Cary, NC, USA).

## 3. Results

### 3.1. Patient Disposition

For study JVDB, between 14 July 2015 and 18 August 2016, 164 of 205 screened patients were randomly assigned to one of the four ramucirumab monotherapy arms (Figure S1A). Of these, 161 patients in total were treated with ramucirumab at the corresponding dosing regimen. As of data cutoff (18 November 2016), 142 (86.6%) of the randomized patients had discontinued, 56.7% due to disease progression.

For study JVCZ, between 22 October 2015 and 26 January 2017, 245 of 305 screened patients were randomly assigned to receive ramucirumab plus paclitaxel at different dosing regimens (Figure S1B). Of these, 243 received study treatment. As of data cutoff (27 October 2017), 232 (94.7%) of the randomized patients had discontinued, 65.3% due to disease progression.

The baseline characteristics of patients were well balanced across treatment arms in both studies (Table S2). Overall, the majority of patients were male, with measurable disease, and ECOG PS of one; the majority had a diagnosis of gastric adenocarcinoma compared to GEJ adenocarcinoma. The median relative dose intensity was high in both studies, ranging from 94 to 100% for ramucirumab across both studies, and 88.5% for paclitaxel in the JVCZ combination study (Table S3). The median duration of ramucirumab therapy across all treatment arms is shown in Table S3.

### 3.2. Pharmacokinetics

**Figure 1:**
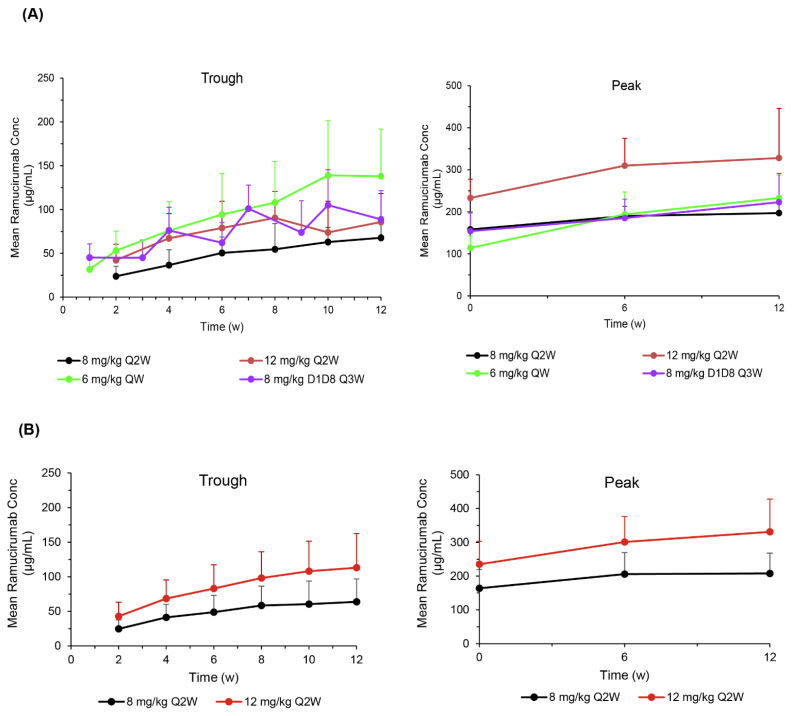
(**A**) JVDB ramucirumab trough and peak serum concentrations (mean ± SD) before and after administration of indicated doses of ramucirumab monotherapy (*n* = 160 patients). (**B**) JVCZ ramucirumab trough and peak serum concentrations before and after administration of ramucirumab 8 or 12 mg/kg in combination with paclitaxel (*n* = 232 patients). Abbreviations: Conc—concentration; D—day; Q2W—every 2 weeks; Q3W—every 3 weeks; QW—weekly; SD—standard deviation; w—weeks.

PK results are presented in Figure 1. Ramucirumab trough concentrations were higher in the 3 EDRs in JVDB (Arms 2–4) than those observed in the standard 8 mg/kg-Q2W regimen (Arm 1). Ramucirumab exposure (peak and trough concentrations) was increased as expected (by ~50%), between 8 and 12 mg/kg (Figure 1A, JVDB and Figure 1B, JVCZ). In JVDB, six patients (6.9%) had one sample positive for anti-ramucirumab antibodies at any time during the study. No patients met the criteria for treatment-emergent antibodies or had neutralizing antibodies. In JVCZ, 18 (9.3%) patients had samples positive for anti-ramucirumab antibodies of the 194 with evaluable samples. In the 12 mg/kg-Q2W ramucirumab arm, two patients met the criteria for treatment-emergent antibodies; one patient met these criteria in the 8 mg/kg-Q2W ramucirumab arm. No neutralizing antibodies were detected.

### 3.3. Clinical Outcomes

**Figure 2:**
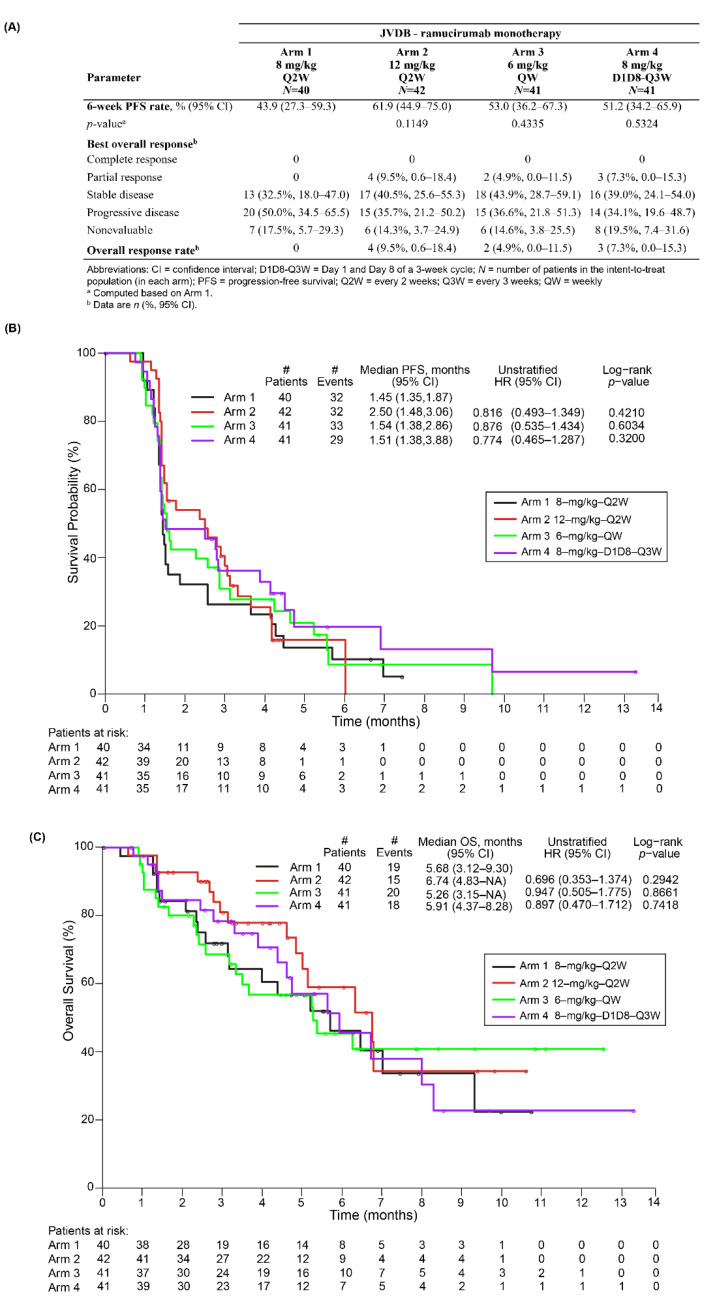
Six-week PFS and objective responses (**A**), PFS (**B**), and OS (**C**) for the standard regimen (Arm 1) and the 3 EDRs (Arms 2, 3 and 4) of ramucirumab monotherapy (JVDB). Abbreviations: EDRs—exploratory dosing regimens; OS—overall survival; PFS—progression-free survival.

In the JVDB monotherapy study, the 6-week PFS rate ranged from 43.9% in Arm 1 (currently registered 8 mg/kg-Q2W dosing regimen) to 61.9% in Arm 2 (12 mg/kg-Q2W), but it was not significantly different between any of the three EDRs and Arm 1 (Figure 2A). The median PFS and OS were approximately 1 month longer in the ramucirumab 12 mg/kg-Q2W regimen compared with Arm 1 (Figure 2B,C). Post-discontinuation therapies for each arm are summarized in Table S4. No complete or partial responses were observed in the 8 mg/kg-Q2W regimen, while nine patients across the three EDRs had a best overall response of partial response, with the 12 mg/kg-Q2W regimen displaying the higher ORR (9.5%). The number of patients with stable disease was also numerically superior for the three EDRs compared with the 8 mg/kg-Q2W arm (Figure 2A).

**Figure 3:**
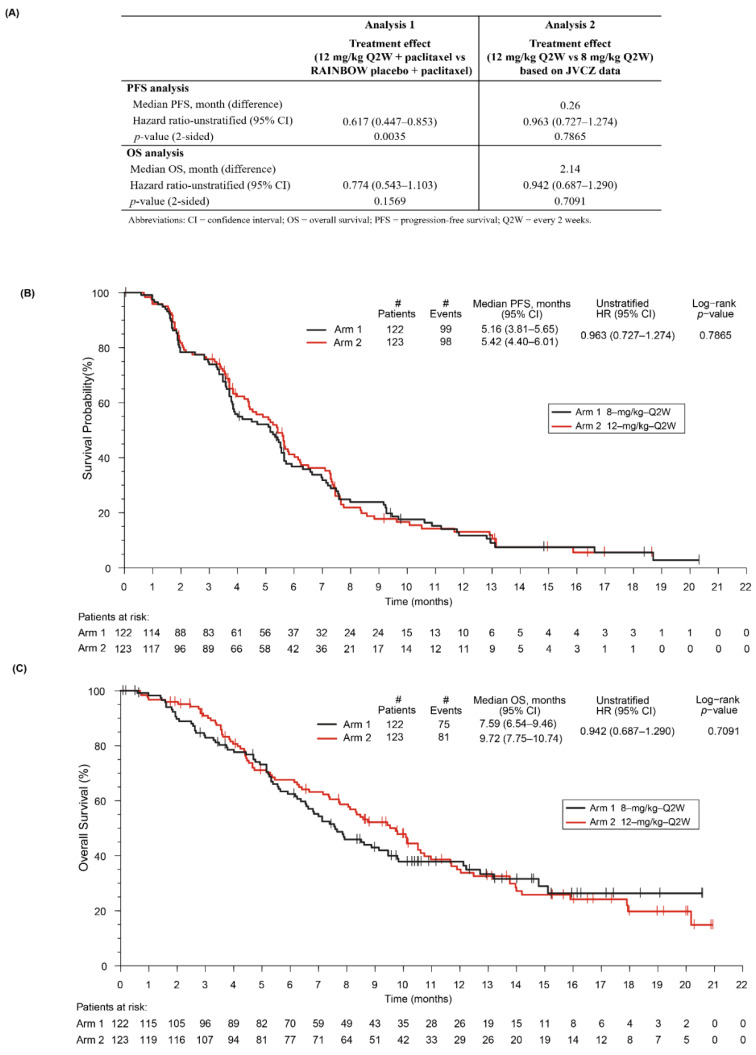
PFS and OS analysis of ramucirumab 12 mg/kg plus paclitaxel in JVCZ versus placebo plus paclitaxel in RAINBOW (Analysis 1) and 12 mg/kg versus 8 mg/kg within JVCZ (Analysis 2) (**A**), PFS (**B**), and OS (**C**). Abbreviations: OS—overall survival; PFS—progression-free survival.

**Figure 4:**
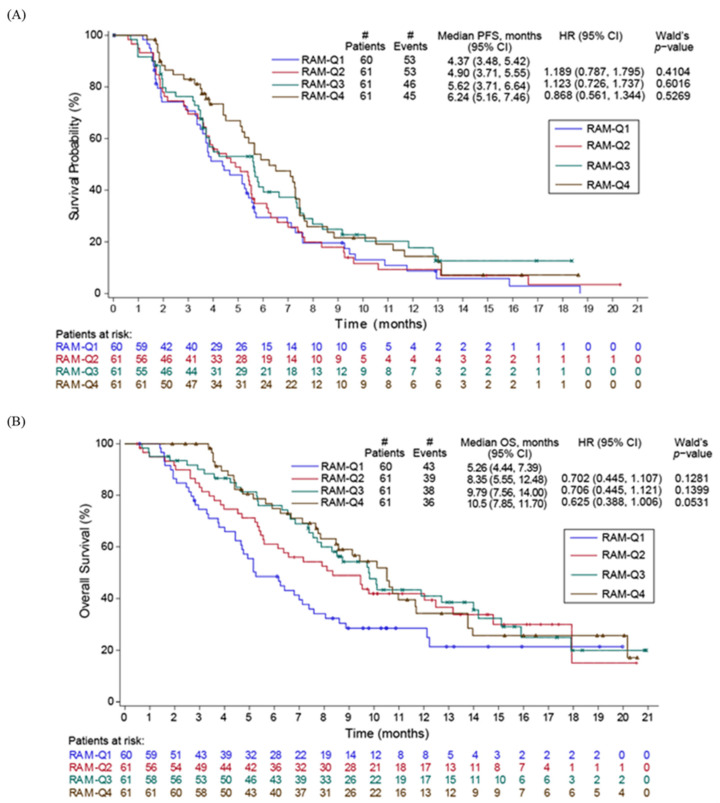
PFS (**A**) and OS (**B**) of JVCZ safety population by ramucirumab C_min,1_ quartiles. Abbreviations: C_min,1_—serum minimum concentration; OS—overall survival; PFS—progression-free survival.

In the JVCZ study, we observed a statistically significant treatment effect on PFS in the ramucirumab 12 mg/kg plus paclitaxel combination arm when compared to the placebo plus paclitaxel arm of RAINBOW [5] using meta-analysis (PFS Analysis 1, *p*-value = 0.0035, Figure 3A), which was our primary endpoint. The same was not observed for the exploratory analysis of OS comparison, which was anticipated since study JVCZ was not powered to show a difference in OS (Analysis 1). In addition, median PFS and OS were not significantly different between the 12 mg/kg and the 8 mg/kg arms using JVCZ data alone (Analysis 2, Figure 3A), despite OS being numerically longer in the ramucirumab 12 mg/kg combination arm (9.72 months vs. 7.59 months) (Figure 3C). Median PFS was 5.42 months (95% CI 4.40, 6.01) and 5.16 months (95% CI 3.81, 5.65) in the ramucirumab 12 and 8 mg/kg plus paclitaxel combination arms, respectively (Figure 3B). Analysis of PFS and OS data by ramucirumab serum minimum concentration (C_min,1_) quartiles in our patient population treated with ramucirumab plus paclitaxel (Figure 4) did not show a statistically significant difference in survival in subsets of patients displaying higher ramucirumab serum concentrations, although this was not a powered analysis. Confirmed ORR and DCR were similar between the two JVCZ treatment arms (Table S5).

### 3.4. Safety

In the JVDB study, treatment-emergent adverse events (TEAEs) occurred in 131 patients (81.4%), of whom 69 patients (42.9%) had TEAEs that were considered related to study treatment (Table S6). The incidence of any grade or grade ≥3 TEAEs was similar among the four treatment arms. More patients reported TEAEs in the ramucirumab 6 mg/kg-QW arm, and more serious AEs (SAEs) were observed in the ramucirumab 8 mg/kg-D1D8-Q3W dosing regimen compared to the 8 mg/kg-Q2W regimen (Table S6). A detailed listing of TEAEs and SAEs reported by treatment arm and preferred term is provided in Table 1. Of note, grade ≥3 fatigue was reported in six patients (14.3%) on the 12 mg/kg-Q2W treatment arm; no other grade ≥3 TEAE was reported in more than 10% of patients on any dosing regimen. Two patients died in the 6 mg/kg-QW arm because of SAEs that were deemed related to ramucirumab: one from gastric hemorrhage and one from respiratory failure (Table 1 and Table S6).

AEs of special interest (AESIs) for ramucirumab, regardless of causality, occurred in 69 patients (42.9%) in the JVDB study. The most common AESIs reported in ≥5% of patients were bleeding/hemorrhage events (19.3%, 31 patients), hypertension (11.8%, 19 patients), and liver injury events (8.7%, 14 patients, mostly aspartate aminotransferase increased) and proteinuria (5.6%, 9 patients), Table S7. The 6 mg/kg-QW dosing regimen had the highest percentage of patients with any grade bleeding/hemorrhage events (34.1%, 14 patients), the most common of which was epistaxis, predominantly of grade 1 or 2 severity. There were two fatal events of gastric hemorrhage reported as the primary cause of death.

In the JVCZ study, TEAEs occurred in 243 patients (96.3%), with 210 (86.4%) experiencing TEAEs deemed related to study treatment (Table S6). Grade 3 or worse TEAEs occurred in 167 patients (68.7%), with similar incidence between the two treatment arms. A detailed listing of TEAEs and SAEs by treatment arm and preferred term is provided in Table 2. SAEs were reported at a higher rate in the 12 mg/kg ramucirumab arm compared to the 8 mg/kg ramucirumab arm (38.2 vs. 25.8%), and discontinuation of study treatment because of AEs was also higher (18.7 vs. 9.2%) (Table S6). Incidence of death due to AEs while on study treatment or within 30 days of treatment discontinuation was the same for both arms (7.3 vs. 6.7%) (Table S6). A higher number of patients reported the SAE of neutropenia in the 12 mg/kg-Q2W ramucirumab arm compared with the 8 mg/kg-Q2W arm (4.9 vs. 0.8%), but the incidence of febrile neutropenia was comparable between arms (3.3 vs. 2.5%) (Table 2). There was a similar rate of deaths attributed to SAEs related to study treatment while on therapy in both arms: five (4.1%) in the 12 mg/kg-Q2W ramucirumab arm (general physical health deterioration [*n* = 2], esophageal fistula, shock hemorrhagic, and tumor perforation) and four patients (3.3%) in the 8 mg/kg-Q2W arm (bone marrow failure, pneumonitis, respiratory distress, and upper gastrointestinal hemorrhage).

In the JVCZ study, AESIs for ramucirumab occurred regardless of causality in 145 patients (59.7%). The most common AESIs reported in ≥2% of patients were epistaxis, hypertension, and aspartate aminotransferase increase (Table S7). The incidence of most AESIs was similar across treatment arms, except for liver-related events, where a higher number of patients reported any grade and grade ≥3 liver-related events in the 12 mg/kg-Q2W ramucirumab arm versus the 8 mg/kg-Q2W arm (Table S7). This was largely due to alanine aminotransferase and aspartate aminotransferase increase, occurring mostly at grade 1–2 severity.

## 4. Discussion

JVDB and JVCZ both examined whether higher doses of ramucirumab as monotherapy or in combination with paclitaxel would improve efficacy while maintaining a favorable safety profile in patients with advanced gastric/GEJ adenocarcinoma. The dosing regimen of ramucirumab used in the 3 EDRs of the JVDB and JVCZ trials differs from the approved dose (8 mg/kg-Q2W). The rationale for these studies was based on exploratory exposure–response analyses of prior trials, suggesting higher exposure might achieve better outcomes [8,9]. For the dosing regimens included in the current studies (8- and 12 mg/kg-Q2W), similar serum ramucirumab concentrations were observed between the JVCZ and JVDB respective dosing regimens. Exposure achieved with the 8 mg/kg-Q2W dosing regimen in JVDB and JVCZ was consistent with that observed in REGARD and RAINBOW phase III studies [4,5]. In JVDB, higher ramucirumab trough concentrations were associated with the three EDRs compared to the currently registered 8 mg/kg-Q2W dosing regimen. In JVCZ, ramucirumab exposure also increased as expected (by ~50%) in the 12 mg/kg-Q2W arm compared to the 8 mg/kg-Q2W regimen.

The safety profile of ramucirumab observed in these studies was consistent with the established safety profile reported in REGARD and RAINBOW trials. In JVDB, the incidence of most AE categories was similar across all four dosing regimens, though the occurrence of SAEs was slightly higher in the experimental ramucirumab 8 mg/kg-D1D8-Q3W arm. In addition, the 6 mg/kg-QW dosing regimen had higher incidences of bleeding/hemorrhage events, including fatal gastrointestinal hemorrhage events. This could have been due to the slightly higher ramucirumab trough concentrations at some time points in these experimental arms. Our conclusions on the effect of alternative dose timings on safety are limited by the small sample size and high standard deviation. Nevertheless, the safety profile described here for these ramucirumab-specific AEs is consistent with that described in the meta-analysis of six randomized trials in 4996 patients [10]. In the JVCZ ramucirumab 12 mg/kg-Q2W plus paclitaxel arm, more patients experienced SAEs and discontinuation of study treatment due to AEs compared with the 8 mg/kg-Q2W arm, but the rates of these events are in line with that observed previously [5]. Our results demonstrate that no clinically meaningful additional toxicity was observed with alternative doses of ramucirumab as a single agent or in combination with paclitaxel when compared with the currently registered ramucirumab regimen of 8 mg/kg-Q2W in patients with gastric/GEJ adenocarcinoma.

In JVDB, the hazard ratios for the 12 mg/kg-Q2W and 8 mg/kg-D1D8–Q3W dosing regimens suggest a potential trend toward improved PFS and OS compared with the currently registered ramucirumab 8 mg/kg-Q2W dose. However, this study was not powered for statistical comparisons on efficacy parameters due to the small size of the arms, preventing us from making additional inferences. Additional studies would be needed to confirm these results in a higher number of patients.

The JVCZ study met its primary objective, demonstrating a statistically significant treatment effect on PFS with ramucirumab 12 mg/kg plus paclitaxel versus the placebo plus paclitaxel arm from RAINBOW. Ramucirumab exposure in the 12 mg/kg regimen was increased by 50% from that achieved with the 8 mg/kg regimen; nevertheless, no statistically significant difference was observed in terms of PFS between these two regimens, which was our secondary objective. The safety data from JVCZ are consistent with that previously reported for RAINBOW [5].

Overall, our results demonstrate no statistically significant additional survival benefit associated with a higher dose and exposure of ramucirumab as a single agent or in combination with paclitaxel compared with the current approved dose of 8 mg/kg-Q2W. Furthermore, there was no meaningful additional toxicity, likely because the doses were still far below the MTD of 13 mg/kg/week [2]. The safety profile appeared consistent across EDRs and with the previously observed safety profile of ramucirumab in gastric/GEJ adenocarcinoma. This is despite prior exposure-response analyses suggesting a positive relationship between ramucirumab exposure and survival [8,9], leading us to hypothesize that an improved benefit could be achieved by increasing the dose to 12 mg/kg. A similar lack of clinically relevant exposure-efficacy benefit has been reported for other antineoplastic monoclonal antibodies (mAbs), such as pembrolizumab in melanoma and non-small cell lung cancer [12] and trastuzumab in metastatic gastric adenocarcinoma, where higher concentrations did not translate into increased survival [13]. A pooled analysis of ipilimumab phase II and III data in melanoma also showed no benefit with different dosing [14] despite initial results suggesting an improvement in OS at the expense of safety [15,16]. The underlying mechanisms for this lack of dose dependency are unclear but several hypotheses have been suggested, including a correlation between cachexia and mAb catabolism [12]. On the other hand, a dose-response effect translating into higher efficacy with a higher dose of bevacizumab has been reported in a randomized phase II trial of bevacizumab in combination with chemotherapy for non-small cell lung cancer [17].

A population PK model for ramucirumab has been developed [18], and none of the clinical factors known to be prognostic for response in gastric cancer (e.g., ECOG status, presence of metastatic sites) were found to be significantly correlated to PK parameters or exposure. Consequently, the lack of an exposure-efficacy relationship for study JVCZ is unlikely to be biased by a confounding relationship between efficacy prognostic factors and PK. The exposure-response analysis presented here did not establish that increasing the dose from 8 to 12 mg/kg improved efficacy.

## 5. Conclusions

Given the lack of a statistically significant greater benefit seen with an increase of up to 50% in doses of ramucirumab, both as monotherapy or in combination with paclitaxel in the JVDB and JVCZ studies, we conclude the approved intravenous ramucirumab dosing regimen of 8 mg/kg Q2W is clinically appropriate for the second-line treatment of adult patients with advanced or metastatic gastric/GEJ adenocarcinoma.

## Figures and Tables

**Table 1:** Study JVDB: treatment-emergent adverse events and serious adverse events.

TEAEs (All-Grade) Reported in ≥10% of Patients by MedDRA Preferred Term ^a^	Number (%) of Patients (JVDB)				
	Arm 1 8 mg/kg Q2W *N* = 38	Arm 2 12 mg/kg Q2W *N* = 42	Arm 3 6 mg/kg QW *N* = 41	Arm 4 8 mg/kg D1D8-Q3W *N* = 40	Total *N* = 161
Patients with ≥1 TEAE	31 (81.6)	32 (76.2)	36 (87.8)	32 (80.0)	131 (81.4)
*Fatigue*	9 (23.7)	15 (35.7)	10 (24.4)	12 (30.0)	46 (28.6)
Decreased appetite	8 (21.1)	9 (21.4)	11 (26.8)	6 (15.0)	34 (21.1)
*Abdominal pain*	7 (18.4)	12 (28.6)	9 (22.0)	6 (15.0)	34 (21.1)
Vomiting	7 (18.4)	7 (16.7)	7 (17.1)	8 (20.0)	29 (18.0)
Nausea	6 (15.8)	4 (9.5)	4 (9.8)	6 (15.0)	20 (12.4)
Aspartate aminotransferase increased	5 (13.2)	5 (11.9)	2 (4.9)	0 (0.0)	12 (7.5)
Hypertension	5 (13.2)	2 (4.8)	7 (17.1)	5 (12.5)	19 (11.8)
Pyrexia	5 (13.2)	2 (4.8)	4 (9.8)	1 (2.5)	12 (7.5)
Anemia	4 (10.5)	5 (11.9)	5 (12.2)	2 (5.0)	16 (9.9)
Dyspnea	4 (10.5)	4 (9.5)	2 (4.9)	4 (10.0)	14 (8.7)
Headache	4 (10.5)	5 (11.9)	5 (12.2)	3 (7.5)	17 (10.6)
Hypoalbuminemia	4 (10.5)	2 (4.8)	3 (7.3)	0	9 (5.6)
Proteinuria	4 (10.5)	1 (2.4)	2 (4.9)	2 (5.0)	9 (5.6)
Alanine aminotransferase increased	3 (7.9)	5 (11.9)	0	0	8 (5.0)
Diarrhea	3 (7.9)	10 (23.8)	3 (7.3)	5 (12.5)	21 (13.0)
Constipation	2 (5.3)	6 (14.3)	7 (17.1)	7 (17.5)	22 (13.7)
Epistaxis	1 (2.6)	1 (2.4)	5 (12.2)	2 (5.0)	9 (5.6)
Dyspepsia	0	1 (2.4)	0	4 (10.0)	5 (3.1)
**TEAEs (Grade ≥3) reported in ≥5% of patients by MedDRA Preferred Term ^a^**	**Arm 1** **8 mg/kg Q2W** ***N* = 38**	**Arm 2** **12 mg/kg Q2W** ***N* = 42**	**Arm 3** **6 mg/kg QW** ***N* = 41**	**Arm 4** **8 mg/kg D1D8-Q3W** ***N* = 40**	**Total** ***N* = 161**
Patients with ≥1 grade ≥3 TEAE	14 (36.8)	18 (42.9)	18 (43.9)	16 (40.0)	66 (41.0)
Decreased appetite	3 (7.9)	0	0	0	3 (1.9)
Hypertension	3 (7.9)	1 (2.4)	2 (4.9)	2 (5.0)	8 (5.0)
*Abdominal pain*	3 (7.9)	2 (4.8)	2 (4.9)	3 (7.5)	10 (6.2)
Anemia	2 (5.3)	2 (4.8)	2 (4.9)	1 (2.5)	7 (4.3)
Blood alkaline phosphatase increased	2 (5.3)	1 (2.4)	0	0	3 (1.9)
Muscular weakness	2 (5.3)	0	1 (2.4)	0	3 (1.9)
Pneumothorax	2 (5.3)	0	0	0	2 (1.2)
Pyrexia	2 (5.3)	0	0	0	2 (1.2)
*Fatigue*	1 (2.6)	6 (14.3)	1 (2.4)	3 (7.5)	11 (6.8)
Vomiting	1 (2.6)	2 (4.8)	0	3 (7.5)	6 (3.7)
Back pain	0	0	2 (4.9)	2 (5.0)	4 (2.5)
**SAEs reported in ≥2 patients by MedDRA Preferred Term ^a^**	**Arm 1** **8 mg/kg Q2W** ***N* = 38**	**Arm 2** **12 mg/kg Q2W** ***N* = 42**	**Arm 3** **6 mg/kg QW** ***N* = 41**	**Arm 4** **8 mg/kg D1D8-Q3W** ***N* = 40**	**Total** ***N* = 161**
Patients with ≥1 SAE	10 (26.3)	9 (21.4)	10 (24.4)	14 (35.0)	43 (26.7)
Non-cardiac chest pain	2 (5.3)	0	0	0	2 (1.2)
Pyrexia	2 (5.3)	0	0	0	2 (1.2)
Vomiting	1 (2.6)	1 (2.4)	0	3 (7.5)	5 (3.1)
*Abdominal pain*	1 (2.6)	1 (2.4)	0	2 (5.0)	4 (2.5)
Back pain	1 (2.6)	0	1 (2.4)	1 (2.5)	3 (1.9)
Cardiac arrest	1 (2.6)	1 (2.4)	0	0	2 (1.2)
Device related infection	1 (2.6)	1 (2.4)	0	0	2 (1.2)
Pneumothorax	1 (2.6)	0	0	1 (2.5)	2 (1.2)
Dysphagia	0	1 (2.4)	1 (2.4)	1 (2.5)	3 (1.9)
Gastric hemorrhage	0	0	3 (7.3) ^b^	0	3 (1.9)
Pneumonia	0	2 (4.8)	0	1 (2.5)	3 (1.9)
Anemia	0	1 (2.4)	0	1 (2.5)	2 (1.2)
Dyspnea	0	1 (2.4)	1 (2.4)	0	2 (1.2)
Gastrointestinal hemorrhage	0	0	1 (2.4)	1 (2.5)	2 (1.2)
Hematemesis	0	0	1 (2.4)	1 (2.5)	2 (1.2)
Hemoptysis	0	0	1 (2.4)	1 (2.5)	2 (1.2)
Pulmonary embolism	0	1 (2.4)	0	1 (2.5)	2 (1.2)
Respiratory failure	0	0	1 (2.4) ^c^	1 (2.5)	2 (1.2)
Upper gastrointestinal hemorrhage	0	1 (2.4)	1 (2.4)	0	2 (1.2)

Abbreviations: D1D8-Q3W—day 1 and 8 of a 3-week cycle; MedDRA—Medical Dictionary for Regulatory Activities Version 19.1; *N* = number of patients in the safety population; Q2W—every 2 weeks; Q3W—every 3 weeks; QW—weekly; SAE—serious adverse event; TEAE—treatment-emergent adverse event. ^a^ Ordered by decreasing frequency in Arm 1. Italicized items are consolidated terms incorporating the multiple MedDRA preferred terms. ^b^ One patient died in the 6 mg/kg QW arm because of the SAE gastric hemorrhage, deemed related to ramucirumab. ^c^ One patient died in the 6 mg/kg QW arm because of the SAE respiratory failure, deemed related to ramucirumab.

**Table 2:** Study JVCZ: treatment-emergent adverse events and serious adverse events.

TEAEs (All-Grade) Reported in ≥10% of Patients on Either Dose by MedDRA Preferred Term ^a^	Number (%) of Patients (JVCZ)		
	Arm 1 Ramucirumab 8 mg/kg + Paclitaxel 80 mg/m^2 b^ *N* = 120	Arm 2 Ramucirumab 12 mg/kg + Paclitaxel 80 mg/m^2 b^ *N* = 123	Total *N* = 243
Patients with ≥1 TEAE (any grade)	116 (96.7)	118 (95.9)	234 (96.3)
*Fatigue*	63 (52.5)	58 (47.2)	121 (49.8)
*Neutropenia*	43 (35.8)	52 (42.3)	95 (39.1)
*Neuropathy*	50 (41.7)	37 (30.1)	87 (35.8)
*Anemia*	39 (32.5)	36 (29.3)	75 (30.9)
Diarrhea	35 (29.2)	32 (26.0)	67 (27.6)
*Abdominal pain* ^c^	35 (29.2)	30 (24.4)	65 (26.7)
Nausea	37 (30.8)	28 (22.8)	65 (26.7)
Vomiting	28 (23.3)	31 (25.2)	59 (24.3)
Decreased appetite	31 (25.8)	25 (20.3)	56 (23.0)
Epistaxis	28 (23.3)	26 (21.1)	54 (22.2)
Hypertension	21 (17.5)	24 (19.5)	45 (18.5)
Constipation	21 (17.5)	23 (18.7)	44 (18.1)
Alopecia	24 (20.0)	19 (15.4)	43 (17.7)
*Leukopenia*	20 (16.7)	21 (17.1)	41 (16.9)
Stomatitis	18 (15.0)	15 (12.2)	33 (13.6)
Aspartate aminotransferase increased	8 (6.7)	21 (17.1)	29 (11.9)
Weight decreased	13 (10.8)	14 (11.4)	27 (11.1)
*Thrombocytopenia*	11 (9.2)	15 (12.2)	26 (10.7)
*Hypoalbuminemia*	15 (12.5)	10 (8.1)	25 (10.3)
**TEAEs (Grade ≥3) reported in ≥5% of patients on either dose by MedDRA Preferred Term ^a^**	**Arm 1** **ramucirumab 8 mg/kg + paclitaxel 80 mg/m^2 b^** ***N* = 120**	**Arm 2** **ramucirumab 12 mg/kg + paclitaxel 80 mg/m^2 b^** ***N* = 123**	**Total** ***N* = 243**
Patients with ≥1 Grade ≥3 TEAE	80 (66.7)	87 (70.7)	167 (68.7)
*Neutropenia*	32 (26.7)	39 (31.7)	71 (29.2)
*Fatigue*	19 (15.8)	16 (13)	35 (14.4)
*Anemia*	11 (9.2)	12 (9.8)	23 (9.5)
*Neuropathy*	11 (9.2)	11 (8.9)	22 (9.1)
*Leukopenia*	9 (7.5)	12 (9.8)	21 (8.6)
Hypertension	10 (8.3)	10 (8.1)	20 (8.2)
*Abdominal pain* ^c^	3 (2.5)	11 (8.9)	14 (5.8)
**SAEs reported in ≥2 patients on either dose by MedDRA Preferred Term ^a^**	**Arm 1** **ramucirumab 8 mg/kg + paclitaxel 80 mg/m^2 b^** ***N* = 120**	**Arm 2** **ramucirumab 12 mg/kg + paclitaxel 80 mg/m^2 b^** ***N* = 123**	**Total** ***N* = 243**
Patients with ≥1 SAE	31 (25.8)	47 (38.2)	78 (32.1)
Febrile neutropenia	3 (2.5)	4 (3.3)	7 (2.9)
*Neutropenia*	1 (0.8)	6 (4.9)	7 (2.9)
*Abdominal pain* ^c^	2 (1.7)	4 (3.3)	6 (2.5)
*Anemia*	3 (2.5)	3 (2.4)	6 (2.5)
*Fatigue*	1 (0.8)	3 (2.4)	4 (1.6)
Gastric hemorrhage	1 (0.8)	3 (2.4)	4 (1.6)
Vomiting	1 (0.8)	3 (2.4)	4 (1.6)
Ascites	1 (0.8)	1 (0.8)	2 (0.8)
Back pain	1 (0.8)	1 (0.8)	2 (0.8)
Blood bilirubin increased	0	2 (1.6)	2 (0.8)
Diarrhea	1 (0.8)	1 (0.8)	2 (0.8)
Dyspnea	1 (0.8)	1 (0.8)	2 (0.8)
Gastrointestinal hemorrhage	0	2 (1.6)	2 (0.8)
General physical health deterioration	0	2 (1.6)	2 (0.8)
Ileus	2 (1.7)	0	2 (0.8)
Infection	0	2 (1.6)	2 (0.8)
Jaundice	0	2 (1.6)	2 (0.8)
Lung infection	1 (0.8)	1 (0.8)	2 (0.8)
Pneumothorax	0	2 (1.6)	2 (0.8)
Small intestinal obstruction	1 (0.8)	1 (0.8)	2 (0.8)
Upper gastrointestinal hemorrhage	1 (0.8)	1 (0.8)	2 (0.8)

Abbreviations: MedDRA—Medical Dictionary for Regulatory Activities Version 20.1; *N*—number of patients in the safety population; SAE—serious adverse event; TEAE—treatment-emergent adverse event. ^a^ Italicized items are consolidated terms incorporating the multiple MedDRA preferred terms. ^b^ Ramucirumab was administered on Days 1 and 15 of each 28-day cycle. Paclitaxel was administered on Days 1, 8, and 15 of each 28-day cycle. ^c^ As used in this table, “abdominal pain” includes the MedDRA preferred terms of “abdominal pain”, “abdominal pain upper”, as well as “gastrointestinal pain” and “abdominal pain lower”.

## Data Availability

Eli Lilly and Company provides access to all individual participant data collected during the trial, after anonymization, with the exception of pharmacokinetic or genetic data. Data are available to request 6 months after the indication studied has been approved in the USA and EU and after primary publication acceptance, whichever is later. No expiration date of data requests is currently set once data are made available. Access is provided after a proposal has been approved by an independent review committee identified for this purpose and after receipt of a signed data sharing agreement. Data and documents, including the study protocol, statistical analysis plan, clinical study report, blank or annotated case report forms, will be provided in a secure data sharing environment. For details on submitting a request, see the instructions provided at www.vivli.org (accessed on 22 December 2021).

## Supplementary Materials

The following supporting information can be downloaded at: https://www.mdpi.com/article/10.3390/cancers14051168/s1, Online Supplementary Methods S1: Full inclusion/exclusion criteria for studies JVDB and JVCZ, Online Supplementary Methods S2: Efficacy measures for studies JVDB and JVCZ; Figure S1: CONSORT diagrams. (A) Study I4T-MC-JVDB (B) Study I4T-MC-JVCZ; Table S1: Ramucirumab dosing and pharmacokinetic and immunogenicity sampling schedule, Table S2: Combined demographics and disease characteristics of patients randomized to ramucirumab monotherapy (JVDB) or combination therapy (JVCZ), Table S3: Summary of drug exposure (safety population) for studies JVDB and JVCZ, Table S4: Summary of post-treatment discontinuation therapies received by patients in studies JVDB and JVCZ (by arm), Table S5: Best overall response for JVCZ study per RECISTv1.1 in solid tumors, Table S6: Safety overview (JVDB/JVCZ), Table S7: Treatment-emergent AESIs for ramucirumab (regardless of causality) reported in the safety population of (A) ramucirumab monotherapy JVDB, and (B) combination therapy JVCZ studies.

## References

[1] (2020). CYRAMZA [package insert USA].

[2] Spratlin J.L., Cohen R.B., Eadens M., Gore L., Camidge D.R., Diab S., Leong S., O’Bryant C., Chow L.Q.M., Serkova N.J. (2010). Phase I pharmacologic and biologic study of ramucirumab (IMC-1121B), a fully hu-man immunoglobulin G1 monoclonal antibody targeting the vascular endothelial growth factor receptor-2. J. Clin. Oncol..

[3] Garon E.B., Ciuleanu T.-E., Arrieta O., Prabhash K., Syrigos K.N., Goksel T., Park K., Gorbunova V., Kowalyszyn R.D., Pikiel J. (2014). Ramucirumab plus docetaxel versus placebo plus docetaxel for second-line treat-ment of stage IV non-small-cell lung cancer after disease progression on platinum-based therapy (REVEL): A multicentre, double-blind, randomised phase 3 trial. Lancet.

[4] Fuchs C.S., Tomasek J., Yong C.J., Dumitru F., Passalacqua R., Goswami C., Safran H., Vieira Dos Santos L., Aprile G., Ferry D.R. (2014). REGARD Trial Investigators. Ramucirumab monotherapy for previously treated advanced gastric or gas-tro-oesophageal junction adenocarcinoma (REGARD): An international, randomised, multicentre, placebo-controlled, phase 3 trial. Lancet.

[5] Wilke H., Muro K., Van Cutsem E., Oh S.C., Bodoky G., Shimada Y., Hironaka S., Sugimoto N., Lipatov O., Kim T.-Y. (2014). Ramucirumab plus paclitaxel versus placebo plus paclitaxel in patients with previ-ously treated advanced gastric or gastro-oesophageal junction adenocarcinoma (RAINBOW): A double-blind, randomised phase 3 trial. Lancet Oncol..

[6] Zhu A.X., Kang Y.K., Yen C.J., Finn R.S., Galle P.R., Llovet J.M., REACH-2 Study Investigators (2019). Ramucirumab after sorafenib in patients with advanced hepatocellular carcinoma and in-creased alpha-fetoprotein concentrations (REACH-2): A randomised, double-blind, placebo-controlled, phase 3 trial. Lancet Oncol..

[7] Petrylak D.P., De Wit R., Chi K.N., Drakaki A., Sternberg C.N., Nishiyama H., Vaishampayan U. (2017). Ramucirumab plus docetaxel versus placebo plus docetaxel in patients with locally advanced or metastatic urothelial carcinoma after platinum-based therapy (RANGE): A randomised, double-blind, phase 3 trial. Lancet.

[8] Tabernero J., Ohtsu A., Muro K., Van Cutsem E., Oh S.C., Bodoky G., Shimada Y., Hironaka S., Ajani J.A., Tomasek J. (2017). Exposure-Response Analyses of Ramucirumab from Two Randomized, Phase III Trials of Second-line Treatment for Advanced Gastric or Gastroesophageal Junction Cancer. Mol. Cancer Ther..

[9] Jin R., Li H., Zhang L.H., Zhao H., Fashoyin-Aje L., Lemery S., Keegan P., Booth B., Rahman N.A., Wang Y. (2015). Exposure-response (E-R) and case-control analyses of ramucirumab leading to recommendation for dosing optimization in patients with gastric cancer [abstract]. J. Clin. Oncol..

[10] Eisenhauer E.A., Therasse P., Bogaerts J., Schwartz L.H., Sargent D., Ford R., Dancey J., Arbuck S., Gwyther S., Mooney M. (2009). New response evaluation criteria in solid tumours: Revised RECIST guideline (version 1.1). Eur. J. Cancer.

[11] Arnold D., Fuchs C.S., Tabernero J., Ohtsu A., Zhu A.X., Garon E.B., Mackey J.R., Paz-Ares L., Baron A.D., Okusaka T. (2017). Meta-analysis of individual patient safety data from six randomized, place-bo-controlled trials with the antiangiogenic VEGFR2-binding monoclonal antibody ramucirumab. Ann. Oncol..

[12] Turner D.C., Kondic A.G., Anderson K.M., Robinson A.G., Garon E.B., Riess J.W., Jain L., Mayawala K., Kang J., Ebbinghaus S.W. (2018). Pembrolizumab Exposure–Response Assessments Challenged by Association of Cancer Cachexia and Catabolic Clearance. Clin. Cancer Res..

[13] Shah M.A., Xu R.H., Bang Y.J., Hoff P.M., Liu T., Herráez-Baranda L.A., Xia F., Garg A., Shing M., Tabernero J. (2017). HELOISE: Phase IIIb randomized multicenter study comparing standard-of-care and high-er-dose trastuzumab regimens combined with chemotherapy as first-line therapy in patients with human epidermal growth factor receptor 2-positive metastatic gastric or gastroesophageal junction adenocarcinoma. J. Clin. Oncol..

[14] Schadendorf D., Hodi F.S., Robert C., Weber J., Margolin K., Hamid O., Patt D., Chen T.-T., Berman D.M., Wolchok J.D. (2015). Pooled Analysis of Long-Term Survival Data From Phase II and Phase III Trials of Ipilimumab in Unresectable or Metastatic Melanoma. J. Clin. Oncol..

[15] Ascierto P.A., Del Vecchio M., Robert C., Mackiewicz A., Chiarion-Sileni V., Arance A., Lebbé C., Bastholt L., Hamid O., Rutkowski P. (2017). Ipilimumab 10 mg/kg versus ipilimumab 3 mg/kg in patients with unresectable or metastatic melanoma: A randomised, double-blind, multicentre, phase 3 trial. Lancet Oncol..

[16] Menzies A.M., Long G.V. (2017). Optimum dosing of ipilimumab in melanoma: Too little, too late?. Lancet Oncol..

[17] Johnson D.H., Fehrenbacher L., Novotny W.F., Herbst R.S., Nemunaitis J.J., Jablons D.M., Langer C.J., Devore R.F., Gaudreault J., Damico L.A. (2004). Randomized Phase II Trial Comparing Bevacizumab Plus Carboplatin and Paclitaxel With Carboplatin and Paclitaxel Alone in Previously Untreated Locally Advanced or Metastatic Non-Small-Cell Lung Cancer. J. Clin. Oncol..

[18] O’Brien L., Westwood P., Gao L., Heathman M. (2017). Population pharmacokinetic meta-analysis of ramucirumab in cancer patients. Br. J. Clin. Pharmacol..

