# Supplementary material for: Evaluating Alternative Ramucirumab Doses as a Single Agent or with Paclitaxel in Second-Line Treatment of Locally Advanced or Metastatic Gastric/Gastroesophageal Junction Adenocarcinoma: Results from Two Randomized, Open-Label, Phase II Studies
Source: Cancers (Basel). 2022 Feb 24;14(5):1168. doi: 10.3390/cancers14051168 (PMC8909008; doi:10.3390/cancers14051168)
Supplement: Supplementary file 1 [file cancers-14-01168-s001.zip › cancers-1545460-supplementary.pdf]

Online Supplementary Methods S1. Full inclusion/Exclusion criteria for studies JVDB and JVCZ

I. JVDB

JVDB Inclusion Criteria

Patients are eligible to be included in the study only if they meet **all** of the following criteria:

[1] The patient has a histopathologically or cytologically confirmed diagnosis of gastric or gastroesophageal junction (GEJ) (Siewert Types I-III) adenocarcinoma.

[2] The patient has documented disease progression during or within 4 months after the last dose of first-line chemotherapy for metastatic disease, or during or within 6 months after the last dose of neoadjuvant or adjuvant therapy.

a. Elevations in carcinoembryonic antigen or other tumor markers without radiographic evidence of progression do not constitute satisfactory evidence of progression on prior therapy.

b. Patients who are intolerant to first-line chemotherapy regimens are eligible, provided disease progression was assessed within 4 months after the last dose of first-line therapy.

[3] The patient received combination chemotherapy prior to disease progression.

a. Prior chemotherapy regimens must include a platinum and/or a fluoropyrimidine component and must not include an antiangiogenic agent (either approved or experimental treatment). Exposure to antineoplastic therapy in addition to platinums and/or fluoropyrimidines is acceptable if the agents were used in the first-line metastatic or neoadjuvant/adjuvant setting.

b. Patients who have had 1 or more components of first-line chemotherapy discontinued because of toxicity, but continued to receive the other component(s), are eligible following disease progression.

[4] The patient has metastatic disease or locally advanced and unresectable disease that is measurable, or nonmeasurable but evaluable, by radiological imaging per Response Evaluation Criteria in Solid Tumors, Version 1.1 (RECIST 1.1) (Eisenhauer et al. 2009). Baseline tumor assessment should be performed using a high-resolution computed tomography (CT) scan using intravenous and oral contrast unless clinically contraindicated. Magnetic resonance imaging (MRI) is acceptable if a CT cannot be performed.

[5] Patients are eligible if they are considered not appropriate, for whatever reason, for treatment with ramucirumab in combination with paclitaxel.

[6] The patient has an Eastern Cooperative Oncology Group (ECOG) performance status of 0 or 1.

[7] The patient has adequate organ function, including:

a. Total bilirubin ≤1.5 × the upper limit of institutional normal (ULN) and alanine aminotransferase (ALT) and aspartate aminotransferase (AST) ≤3 × ULN. If the liver has tumor involvement, AST, and ALT <5 × ULN are acceptable.

b. Serum creatinine ≤1.5 × ULN or calculated creatinine clearance (per the Cockcroft-Gault formula or equivalent and/or 24-hour urine collection) ≥60 mL/min.

c. Urinary protein is <2+ on dipstick or routine urinalysis.

If urine dipstick or routine urinalysis indicates proteinuria ≥2+, then a 24-hour urine collection must be performed and must demonstrate urine protein <2 g to allow the patient to participate in the study.

d. Absolute neutrophil count ≥1.5 × 10^9/L, platelets ≥100 × 10^9/L, and hemoglobin ≥9 g/dL (5.58 mmol/L). Packed red blood cell transfusions are not allowed within 1 week prior to baseline hematology profile.

e. International normalized ratio (INR) ≤1.5 × ULN and partial thromboplastin time ≤5 seconds above ULN, unless the patient is receiving anticoagulation therapy. Patients on full-dose anticoagulation therapy must be on a stable dose of oral anticoagulation therapy or low molecular weight heparin for a minimum of 14 days. Patients receiving warfarin must have an INR ≤3.0 × ULN and have no bleeding within 14 days prior to the first dose of ramucirumab or pathological condition that carries a high risk of bleeding, such as tumors involving major vessels or known varices.

[8] The patient is at least 18 years old (or of an acceptable age according to local regulations, whichever is older).

[9] The patient has provided written informed consent prior to any study-specific procedures and is amenable to compliance with protocol schedules and testing.

[10] The patient has an estimated life expectancy of ≥12 weeks in the judgment of the investigator.

[11] The patient has resolution to Grade ≤1 by Common Terminology Criteria for Adverse Events (CTCAE) Version 4.0 (NCI 2009), of all clinically significant toxic effects of previous anticancer therapy. Patients with nonserious and nonlife-threatening toxicities, such as alopecia, altered taste, or nail changes, can be considered. Stable Grade 2 neuropathy is permitted.

[12] The patient, if male, is sterile (including vasectomy confirmed by post-vasectomy semen analysis) or agrees to use a reliable method of birth control and to not donate sperm during the study and for at least 12 weeks following the last dose of study treatment.

[13] The patient, if female, is surgically sterile, is postmenopausal, or agrees to use a highly effective method of birth control during the study and for 12 weeks following the last dose of study treatment.

A "highly effective method of birth control" is defined as 1 that results in a low failure rate (that is, <1% per year) when used consistently and correctly.

[14] The patient, if female and of child-bearing potential, must have a negative serum or urine pregnancy test within 7 days prior to randomization.

JVDB Exclusion Criteria

Patients will be excluded from the study if they meet **any** of the following criteria:

[15] The patient has squamous cell or undifferentiated gastric cancer.

[16] The patient is receiving chronic therapy with any of the following within 7 days prior to randomization:

a. Nonsteroidal anti-inflammatory agents (NSAIDs; such as indomethacin, ibuprofen, naproxen, or similar agents), or

b. Other anti-platelet agents (such as clopidogrel, ticlopidine, dipyridamole, or anagrelide).

Aspirin use at doses up to 325 mg/day is permitted.

[17] The patient received radiotherapy within 14 days prior to randomization. Palliative radiotherapy during the study, if clinically indicated, can be considered after consultation with the Lilly clinical research physician. Any lesion requiring palliative radiotherapy, or which has been previously irradiated cannot be considered for response assessment.

[18] The patient received >1 line of prior therapy for the treatment of locally advanced and unresectable or metastatic gastric or GEJ (Siewert Types I-III) adenocarcinoma.

[19] The patient received previous systemic chemotherapy with a cumulative dose of >900 mg/m^2 of epirubicin or >400 mg/m^2 of doxorubicin.

[20] The patient received previous treatment with agents targeting the VEGF/VEGF receptor 2 signaling pathway, including previous exposure to ramucirumab.

[21] The patient has documented brain metastases, leptomeningeal disease, or uncontrolled spinal cord compression. Screening of asymptomatic patients is not required.

[22] The patient has a significant bleeding disorder or vasculitis or had a Grade ≥3 bleeding episode within 12 weeks prior to randomization.

[23] The patient experienced any arterial thromboembolic event, including myocardial infarction, unstable angina, cerebrovascular accident, or transient ischemic attack, within 6 months prior to randomization.

[24] The patient has symptomatic congestive heart failure (CHF; New York Heart Association II-IV) or symptomatic or poorly controlled cardiac arrhythmia.

[25] The patient has uncontrolled hypertension, as defined in CTCAE Version 4.0, prior to initiating study treatment, despite antihypertensive intervention.

CTCAE Version 4.0 defines uncontrolled hypertension as Grade >2 hypertension; clinically, the patient continues to experience elevated blood pressure (systolic >160 mmHg and/or diastolic >100 mmHg) despite medications.

[26] The patient underwent major surgery within 28 days prior to randomization or central venous access device placement within 7 days prior to randomization.

[27] The patient plans to undergo elective major surgery during the course of the trial.

[28] The patient has a history of gastrointestinal (GI) perforation or fistula within 6 months prior to randomization.

[29] The patient has a history of inflammatory bowel disease or Crohn's disease requiring medical intervention (immunomodulatory or immunosuppressive medications or surgery) ≤12 months prior to randomization.

[30] The patient has an acute or subacute bowel obstruction or history of chronic diarrhea that is considered clinically significant in the opinion of the investigator.

[31] The patient has either of the following:

a. Cirrhosis at a level of Child-Pugh B (or worse).

b. Cirrhosis (any degree) and a history of hepatic encephalopathy or clinically meaningful ascites resulting from cirrhosis. Clinically meaningful

ascites is defined as ascites resulting from cirrhosis and requiring ongoing treatment with diuretics and/or paracentesis.

[32] The patient has a known allergy or hypersensitivity to any components of study treatment.

[33] The patient is currently enrolled in a clinical trial involving an investigational product or nonapproved use of a drug or is concurrently enrolled in any other type of medical research judged not to be scientifically or medically compatible with this study. Patients participating in surveys or observational studies are eligible to participate in this study.

[34] The patient received any previous investigational therapy within 4 half-lives of the investigational agent prior to randomization.

[35] The patient has a serious illness or medical condition including, but not limited to, the following:

a. Known human immunodeficiency virus infection or acquired immunodeficiency syndrome-related illness.

b. Active or uncontrolled clinically serious infection.

[36] The patient is pregnant or breastfeeding.

[37] The patient has a concurrent active malignancy other than the following:

a. Adequately treated nonmelanomatous skin cancer.

b. Curatively treated in situ carcinoma of the cervix or other noninvasive carcinoma or in situ neoplasm.

A patient with a history of prior malignancy is eligible if he or she has been disease free for ≥3 years prior to randomization.

[38] The patient has a serious nonhealing: (a) wound, (b) peptic ulcer, or (c) bone fracture, within 28 days prior to randomization.

[39] The patient has unresected primary tumors or local tumor recurrence following resection **and** is receiving anticoagulation therapy.

[40] The patient experienced any Grade 3 or 4 venous thromboembolic event (VTE) that is considered by the investigator to be life-threatening or that is symptomatic and not adequately treated by anticoagulation therapy, within 6 months prior to randomization.

[41] The patient has any condition (for example, psychological, geographical, or medical) that does not permit compliance with the study and follow-up procedures or suggests that the patient is, in the investigator's opinion, not an appropriate candidate for the study.

II. JVCZ

JVCZ Inclusion Criteria

Patients are eligible to be included in the study only if they meet **all** of the following criteria:

[1] The patient has a histopathologically or cytologically confirmed diagnosis of gastric or gastroesophageal junction (GEJ) (Siewert Types I-III) adenocarcinoma.

[2] The patient has documented disease progression during or within 4 months after the last dose of first-line chemotherapy for metastatic disease, or during or within 6 months after the last dose of neoadjuvant or adjuvant therapy.

a. Elevations in carcinoembryonic antigen or other tumor markers without radiographic evidence of progression do not constitute satisfactory evidence of progression on prior therapy.

b. Patients who are intolerant to first-line chemotherapy regimens are eligible, provided disease progression was assessed within 4 months after the last dose of first-line therapy.

[3] The patient received combination chemotherapy prior to disease progression.

a. Prior chemotherapy regimens must include a platinum and/or a fluoropyrimidine component and must not include a taxane or antiangiogenic agent (either approved or experimental treatment). Exposure to antineoplastic therapy, in addition to platinums and/or fluoropyrimidines, is acceptable if the agents were used in the first-line metastatic or neoadjuvant/adjuvant setting.

b. Patients who have had 1 or more components of first-line chemotherapy discontinued because of toxicity, but continued to receive the other component(s), are eligible following disease progression.

[4] The patient has metastatic disease or locally advanced disease that is measurable, or nonmeasurable but evaluable, by radiological imaging per Response Evaluation Criteria in Solid Tumors, Version 1.1 (RECIST 1.1) (Eisenhauer et al. 2009). Baseline tumor assessment should be performed using a high-resolution computed tomography (CT) scan using intravenous and oral contrast unless clinically contraindicated. Magnetic resonance imaging (MRI) is acceptable if a CT cannot be performed.

[5] The patient has an ECOG performance status of 0 or 1.

[6] The patient has adequate organ function, including:

a. Total bilirubin ≤1.5 × the upper limit of institutional normal (ULN) and alanine aminotransferase (ALT) and aspartate aminotransferase (AST) ≤3 × ULN. If the liver has tumor involvement, AST, and ALT <5 × ULN are acceptable.

b. Serum creatinine ≤1.5 × ULN or calculated creatinine clearance (per the Cockcroft-Gault formula or equivalent and/or 24-hour urine collection) ≥50 mL/min.

c. Urinary protein is <2+ on dipstick or routine urinalysis.

If urine dipstick or routine urinalysis indicates proteinuria ≥2+, then a 24-hour urine collection must be performed and must demonstrate urine protein <2 g to allow the patient to participate in the study.

d. Absolute neutrophil count ≥1.5 × 10^9/L, platelets ≥100 × 10^9/L, and hemoglobin ≥9 g/dL (5.58 mmol/L). Packed red blood cell transfusions are not allowed within 1 week prior to baseline hematology profile.

e. International normalized ratio (INR) ≤1.5 × ULN and partial thromboplastin time ≤5 seconds above ULN, unless the patient is receiving anticoagulation therapy. Patients receiving warfarin must be switched to low molecular weight heparin and have achieved stable coagulation profile prior to randomization.

[7] The patient is at least 18 years old (or of an acceptable age according to local regulations, whichever is older).

[8] The patient has provided written informed consent prior to any study-specific procedures and is amenable to compliance with protocol schedules and testing.

[9] The patient has an estimated life expectancy of ≥12 weeks in the judgment of the investigator.

[10] The patient has resolution to Grade ≤1 by Common Terminology Criteria for Adverse Events (CTCAE) Version 4.0 (NCI 2009), of all clinically significant toxic effects of previous anticancer therapy. Patients with nonserious and nonlife-threatening toxicities, such as alopecia, altered taste, or nail changes, can be considered. Stable Grade 2 neuropathy is permitted.

[11] The patient, if male, is sterile (including vasectomy confirmed by post-vasectomy semen analysis) or agrees to use a reliable method of birth control and to not donate sperm during the study and for at least 12 weeks following the last dose of study treatment.

[12] The patient, if female, is surgically sterile, is postmenopausal, or agrees to use a highly effective method of birth control during the study and for 12 weeks following the last dose of study treatment.

A "highly effective method of birth control" is defined as 1 that results in a low failure rate (that is, <1% per year) when used consistently and correctly.

[13] The patient, if female and of child-bearing potential, must have a negative serum or urine pregnancy test within 7 days prior to randomization.

JVCZ Exclusion Criteria

Patients will be excluded from the study if they meet **any** of the following criteria:

[14] The patient has cancer with histology other than adenocarcinoma.

[15] The patient is receiving chronic therapy with any of the following within 7 days prior to randomization:

a. Nonsteroidal anti-inflammatory agents (NSAIDs; such as indomethacin, ibuprofen, naproxen, or similar agents).

b. Other anti-platelet agents (such as clopidogrel, ticlopidine, dipyridamole, or anagrelide).

Aspirin use at doses up to 325 mg/day is permitted.

[16] The patient received radiotherapy within 14 days prior to randomization. Palliative radiotherapy during the study, if clinically indicated, can be considered after consultation with the Lilly clinical research physician. Any lesion requiring palliative radiotherapy or which has been previously irradiated cannot be considered for response assessment.

[17] The patient received >1 line of prior therapy for the treatment of locally advanced and unresectable or metastatic gastric or GEJ (Siewert Types I-III) adenocarcinoma.

[18] The patient received previous systemic chemotherapy with a cumulative dose of >900 mg/m^2 of epirubicin or >400 mg/m^2 of doxorubicin.

[19] The patient received previous treatment with agents targeting the VEGF/VEGF receptor 2 signaling pathway, including previous exposure to ramucirumab.

[20] The patient has documented brain metastases, leptomeningeal disease, or uncontrolled spinal cord compression. Screening of asymptomatic patients is not required.

[21] The patient has a significant bleeding disorder or vasculitis or had a Grade ≥3 bleeding episode within 12 weeks prior to randomization.

[22] The patient experienced any arterial thromboembolic event (ATE), including myocardial infarction, unstable angina, cerebrovascular accident, or transient ischemic attack, within 6 months prior to randomization.

[23] The patient has symptomatic congestive heart failure (CHF; New York Heart Association II-IV) or symptomatic or poorly controlled cardiac arrhythmia.

[24] The patient has uncontrolled hypertension, as defined in CTCAE Version 4.0, prior to initiating study treatment, despite antihypertensive intervention.

CTCAE Version 4.0 defines uncontrolled hypertension as Grade >2 hypertension; clinically, the patient continues to experience elevated blood pressure (systolic >160 mmHg and/or diastolic >100 mmHg) despite medications.

[25] The patient underwent major surgery within 28 days prior to randomization or central venous access device placement within 7 days prior to randomization.

[26] The patient plans to undergo elective major surgery during the course of the trial.

[27] The patient has a history of gastrointestinal (GI) perforation or fistula within 6 months prior to randomization.

[28] The patient has a history of inflammatory bowel disease or Crohn's disease requiring medical intervention (immunomodulatory or immunosuppressive medications or surgery) ≤12 months prior to randomization.

[29] The patient has an acute or subacute bowel obstruction or history of chronic diarrhea that is considered clinically significant in the opinion of the investigator.

[30] The patient has either of the following:

a. Cirrhosis at a level of Child-Pugh B (or worse).

b. Cirrhosis (any degree) and a history of hepatic encephalopathy or clinically meaningful ascites resulting from cirrhosis. Clinically meaningful ascites is defined as ascites resulting from cirrhosis and requiring ongoing treatment with diuretics and/or paracentesis.

[31] The patient has known allergy or hypersensitivity to any components of study treatment.

[32] The patient is currently enrolled in a clinical trial involving an investigational product or nonapproved use of a drug or is concurrently enrolled in any other type of medical research judged not to be scientifically or medically compatible with this study. Patients participating in surveys or observational studies are eligible to participate in this study.

[33] The patient received any previous investigational therapy within 4 half-lives of the investigational agent prior to randomization.

[34] The patient has a serious illness or medical condition including, but not limited to, the following:

a. Known human immunodeficiency virus infection or acquired immunodeficiency syndrome-related illness.

b. Active or uncontrolled clinically serious infection.

[35] The patient is pregnant or breastfeeding.

[36] The patient has a concurrent active malignancy other than the following:

a. Adequately treated nonmelanomatous skin cancer.

b. Curatively treated in situ carcinoma of the cervix or other noninvasive carcinoma or in situ neoplasm.

A patient with a history of prior malignancy is eligible if he or she has been disease free for ≥3 years prior to randomization.

[37] The patient has a serious nonhealing: (a) wound, (b) peptic ulcer, or (c) bone fracture, within 28 days prior to randomization.

[38] The patient experienced any Grade 3 or 4 venous thromboembolic event (VTE) that is considered by the investigator to be life-threatening or that is symptomatic and not adequately treated by anticoagulation therapy, within 6 months prior to randomization.

[39] The patient has any condition (for example, psychological, geographical, or medical) that does not permit compliance with the study and follow-up procedures or suggests that the patient is, in the investigator's opinion, not an appropriate candidate for the study.

Online Supplementary Methods S2: Efficacy measures for studies JVDB and JVCZ

Progression-free survival (PFS) (by investigator review) was defined from the date of randomization to the date of radiographic documentation of progression (as defined by RECISTv1.1) or the date of death due to any cause, whichever is earlier.

Objective response rate (ORR) was defined as the proportion of randomized patients achieving a best overall response (BOR) of complete response (CR) or partial response (PR).

Disease control rate (DCR) was defined as the proportion of randomized patients achieving a BOR of CR, PR, or stable disease (SD).

Overall survival (OS) was measured from the date of randomization to death from any cause. If the patient was alive at the data inclusion cutoff date for the analysis (or was lost to follow-up), OS was censored on the last date the patient was known to be alive.

Table S1. Ramucirumab dosing and pharmacokinetic and immunogenicity sampling schedule.

**Time (W)**	**0**	**1**	**2**	**3**	**4**	**5**	**6**	**7**	**8**	**9**	**10**	**11**	**12**
Study day	1	8	15	22	29	36	43	50	57	64	71	78	85
Cycle	1				2				3				4
Dose number	1		2		3		4		5		6		7
Regimen													
Arm 1[a]<br>8 mg/kg-Q2W	▲<br>□		Δ		Δ		▲		Δ<br>□		Δ		▲
Arm 2[b]<br>12 mg/kg-Q2W	▲<br>□		Δ		Δ		▲		Δ<br>□		Δ		▲
Dose number	1	2	3	4	5	6	7	8	9	10	11	12	13
Arm 3<br>6 mg/kg-QW	▲<br>□	Δ	Δ	■	Δ	■	▲	■	Δ	■	Δ	■	▲
Cycle	1			2			3			4			5
Dose number	1	2		3	4		5	6		7	8		9
Arm 4<br>8 mg/kg D1D8-Q3W	▲<br>□	Δ		Δ	Δ		▲<br>□	Δ		Δ	Δ		▲

Abbreviations: D1D8-Q3W = Day 1 and Day 8 of a 3-week cycle; PK = pharmacokinetic; Q2W = every 2 weeks; Q3W = every 3 weeks; QW = weekly; W = week; ▲ Scheduled day for ramucirumab infusion, PK pre-dose sample, and 1-hour post end of infusion sample. Δ Scheduled day for ramucirumab infusion and PK pre-dose sample. ■ Scheduled day for ramucirumab infusion. □ Scheduled day for immunogenicity sample. [a] Arm 1 schedule refers to 8 mg/kg Q2W for both studies JVDB (monotherapy) and JVCZ (combination). [b] Arm 2 schedule refers to 12 mg/kg Q2W for both studies JVDB (monotherapy) and JVCZ (combination).

Table S2. Combined demographics and disease characteristics of patients randomized to ramucirumab monotherapy (JVDB) or combination therapy (JVCZ).

Characteristics	JVDB—monotherapy					JVCZ—combination therapy		
	Arm 1 ramucirumab 8 mg/kg Q2W *N* = 40	Arm 2 ramucirumab 12 mg/kg Q2W *N* = 42	Arm 3 ramucirumab 6 mg/kg QW *N* = 41	Arm 4 ramucirumab 8 mg/kg D1D8-Q3W *N* = 41	Total (all arms) *N* = 164	Arm 1 ramucirumab 8 mg/kg + paclitaxel 80 mg/m² *N* = 122	Arm 2 ramucirumab 12 mg/kg + paclitaxel 80 mg/m² *N* = 123	Total (both arms) *N* = 245
Sex, *n* (%)								
Male	29 (72.5)	32 (76.2)	35 (85.4)	32 (78.0)	128 (78.0)	78 (63.9)	83 (67.5)	161 (65.7)
Female	11 (27.5)	10 (23.8)	6 (14.6)	9 (22.0)	36 (22.0)	44 (36.1)	40 (32.5)	84 (34.3)
Age, years median (range)	61.0 (31–85)	59.5 (31–76)	59.0 (24–83)	57.0 (29–83)	59.0 (24–85)	60 (22–83)	60 (25–78)	60 (22–83)
Age group, *n* (%)								
<65 years	27 (67.5)	29 (69.0)	25 (61.0)	30 (73.2)	111 (67.7)	82 (67.2)	83 (67.5)	165 (67.3)
≥65 years	13 (32.5)	13 (31.0)	16 (39.0)	11 (26.8)	53 (32.3)	40 (32.8)	40 (32.5)	80 (32.7)
Race, *n* (%)								
American, Indian or Alaska Native	NC	NC	NC	NC	NC	0	1 (0.8)	1 (0.4)
Asian	1 (2.5)	0	0	1 (2.4)	2 (1.2)	3 (2.5)	0	3 (1.2)
Black or African American	NC	NC	NC	NC	NC	2 (1.6)	2 (1.6)	4 (1.6)
White	32 (80.0)	35 (83.3)	35 (85.4)	33 (80.5)	135 (82.3)	117 (95.9)	119 (96.7)	236 (96.3)
Multiple races	6 (15.0)	7 (16.7)	5 (12.2)	6 (14.6)	24 (14.6)	NC	NC	NC
Missing	1 (2.5)	0	1 (2.4)	1 (2.4)	3 (1.8)	0	1 (0.8)	1 (0.4)
ECOG PS, *n* (%)								
0	13 (32.5)	13 (31.0)	15 (36.6)	13 (31.7)	54 (32.9)	50 (41.0)	44 (35.8)	94 (38.4)
1	26 (65.0)	29 (69.0)	26 (63.4)	28 (68.3)	109 (66.5)	72 (59.0)	79 (64.2)	151 (61.6)
Missing	1 (2.5)	0	0	0	1 (0.6)	0	0	0
Primary tumor								
GEJ adenocarcinoma	12 (30.0)	18 (42.9)	13 (31.7)	16 (39.0)	59 (36.0)	20 (16.4)	26 (21.1)	46 (18.8)
Gastric adenocarcinoma	27 (67.5)	24 (57.1)	28 (68.3)	25 (61.0)	104 (63.4)	101 (82.8)	96 (78.0)	197 (80.4)
Missing or unknown	1 (2.5)	0	0	0	1 (0.6)	1 (0.8)	1 (0.8)	2 (0.8)

Measurable disease								
Yes	34 (85.0)	36 (85.7)	37 (90.2)	39 (95.1)	146 (89.0)	108 (88.5)	102 (82.9)	210 (85.7)
No	6 (15.0)	6 (14.3)	4 (9.8)	2 (4.9)	18 (11.0)	14 (11.5)	21 (17.1)	35 (14.3)
Peritoneal metastases								
Yes	12 (30.0)	12 (28.6)	14 (34.1)	13 (31.7)	51 (31.1)	31 (25.4)	36 (29.3)	67 (27.3)
No	16 (40.0)	9 (21.4)	14 (34.1)	12 (29.3)	51 (31.1)	90 (73.8)	87 (70.7)	177 (72.2)
Missing	12 (30.0)	21 (50.0)	13 (31.7)	16 (39.0)	62 (37.8)	1 (0.8)	0	1 (0.4)
Pathological subtype								
Diffuse	4 (10.0)	5 (11.9)	7 (17.1)	3 (7.3)	19 (11.6)	23 (18.9)	30 (24.4)	53 (21.6)
Intestinal	10 (25.0)	9 (21.4)	8 (19.5)	12 (29.3)	39 (23.8)	34 (27.9)	24 (19.5)	58 (23.7)
Mixed/unknown	25 (62.5)	27 (64.3)	26 (63.4)	26 (63.4)	104 (63.4)	64 (52.5)	69 (56.1)	133 (54.3)
Missing	1 (2.5)	1 (2.4)	0	0	2 (1.2)	1 (0.8)	0	1 (0.4)
Type of prior therapy								
Surgery	20 (50.0)	21 (50.0)	20 (48.8)	25 (61.0)	86 (52.4)	56 (45.9)	71 (57.7)	127 (51.8)
Radiotherapy	4 (10.0)	1 (2.4)	13 (31.7)	10 (24.4)	28 (17.1)	13 (10.7)	16 (13.0)	29 (11.8)
Systemic therapy	38 (95.0)	42 (100.0)	41 (100.0)	41 (100.0)	162 (98.8)	121 (99.2)	123 (100.0)	244 (99.6)
Neoadjuvant	6 (15.0)	8 (19.0)	6 (14.6)	12 (29.3)	32 (19.5)	15 (12.3)	23 (18.7)	38 (15.5)
Adjuvant	5 (12.5)	5 (11.9)	13 (31.7)	9 (22.0)	32 (19.5)	30 (24.6)	24 (19.5)	54 (22.0)
Advanced or metastatic	28 (70.0)	34 (81.0)	30 (73.2)	30 (73.2)	122 (74.4)	91 (74.6)	95 (77.2)	186 (75.9)

Abbreviations: D1D8-Q3W = Day 1 and Day 8 of a 3-week cycle; ECOG PS = Eastern Cooperative Oncology Group performance status; GEJ = gastroesophageal junction; *N* = number of patients in the ITT population; *n* = number of patients in specified category; Q2W = every 2 weeks; Q3W = every 3 weeks; QW = weekly.

Table S3. Summary of drug exposure (safety population) for studies JVDB and JVCZ.

Parameter	JVDB—monotherapy					JVCZ—combination therapy		
	**Arm 1 ramucirumab 8 mg/kg Q2W *N* = 38**	**Arm 2 ramucirumab 12 mg/kg Q2W *N* = 42**	**Arm 3 ramucirumab 6 mg/kg QW *N* = 41**	**Arm 4 ramucirumab 8 mg/kg D1D8-Q3W *N* = 40**	**Total (all arms) *N* = 161**	**Arm 1 ramucirumab 8 mg/kg + paclitaxel Q2W *N* = 120**	**Arm 2 ramucirumab 12 mg/kg + paclitaxel Q2W *N* = 123**	**Total (both arms) *N* = 243**
**Ramucirumab**								
Median number of cycles (IQR)	2.0 (2.0–5.0)	2.0 (2.0–4.0)	2.0 (2.0–4.0)	2.5 (2.0–5.5)	2.0 (2.0–5.0)	4.0 (2.0–7.0)	5.0 (2.0–8.0)	4.0 (2.0–8.0)
Median duration of therapy, weeks (IQR)	6.21 (6.0–17.71)	8.00 (6.0–14.14)	7.00 (5.14–16.00)	8.07 (6.00–17.18)	8.00 (6.0–16.0)	16.79 (9.07–29.50)	20.00 (9.86–32.00)	17.86 (9.57–31.43)
Median relative dose intensity, % (IQR)[a]	100.00 (98.25–100.30)	100.00 (98.87–100.99)	97.95 (91.09–99.78)	99.26 (97.46–100.00)	99.57 (97.30–100.30)	95.50 (87.92–99.92)	94.12 (85.56–99.76)	94.92 (87.54–99.83)
**Paclitaxel**								
Median number of cycles (IQR)	N/A	N/A	N/A	N/A	N/A	4.00 (2.50–7.00)	5.00 (2.00–7.00)	4.00 (2.00–7.00)
Median duration of therapy, weeks (IQR)	N/A	N/A	N/A	N/A	N/A	16.43 (9.50–27.21)	18.86 (9.57–31.43)	17.14 (9.57–28.14)
Median relative dose intensity, % (IQR)[a]	N/A	N/A	N/A	N/A	N/A	88.63 (79.13–97.12)	88.54 (77.38–97.13)	88.63 (78.36–97.13)

Abbreviations: D1D8-Q3W = Day 1 and Day 8 of a 3-week cycle; IQR = interquartile range; *N* = number of subjects in safety population; *n* = number of subjects in the specified category; N/A = not applicable; Q2W = every 2 weeks; Q3W = every 3 weeks; QW = weekly. [a] Relative dose intensity is calculated as (actual amount of drug taken / amount of drug prescribed) *100%.

Table S4. Summary of post-treatment discontinuation therapies received by patients in studies JVDB and JVCZ (by arm).

Parameter	JVDB—monotherapy				JVCZ—combination therapy	
	**Arm 1 ramucirumab 8 mg/kg Q2W *N* = 40**	**Arm 2 ramucirumab 12 mg/kg Q2W *N* = 42**	**Arm 3 ramucirumab 6 mg/kg QW *N* = 41**	**Arm 4 ramucirumab 8 mg/kg D1D8-Q3W *N* = 41**	**Arm 1 ramucirumab 8 mg/kg + paclitaxel 80 mg/m^2 *N* = 120**	**Arm 2 ramucirumab 12 mg/kg + paclitaxel 80 mg/m^2 *N* = 123**
Post-discontinuation therapy, *n* (%)						
Surgical procedure	0 (0.0)	1 (2.4)	1 (2.4)	0 (0.0)	1 (0.8)	4 (3.3)
Radiotherapy	0 (0.0)	2 (4.8)	0 (0.0)	0 (0.0)	3 (2.5)	2 (1.6)
Systemic therapy	4 (10.0)	4 (9.5)	3 (7.3)	5 (12.2)	29 (24.2)	21 (17.1)
Chemotherapy	4 (10.0)	4 (9.5)	3 (7.3)	5 (12.2)	26 (21.7)	20 (16.3)
Investigational drug	0 (0.0)	0 (0.0)	0 (0.0)	0 (0.0)	1 (0.8)	1 (0.8)
Monoclonal antibody	0 (0.0)	0 (0.0)	0 (0.0)	0 (0.0)	3 (2.5)	2 (1.6)

Abbreviations: D1D8-Q3W = Day 1 and Day 8 of a 3-week cycle; *N* = number of patients; Q2W = every 2 weeks; Q3W = every 3 weeks; QW = weekly.

Table S5. Best overall response for JVCZ study per RECISTv1.1 in solid tumors.

Parameter [a]	JVCZ—combination therapy<br>Arm 1<br>ramucirumab 8 mg/kg<br>+ paclitaxel 80 mg/m^2<br>N = 122	 Arm 2<br>ramucirumab 12 mg/kg<br>+ paclitaxel 80 mg/m^2<br>N = 123
Best overall response		
Complete response	1 (0.8%, 0.0–2.4)	0
Partial response	30 (24.6%, 16.9–32.2)	34 (27.6%, 19.7–35.5)
Stable disease	61 (50.0%, 41.1–58.9)	63 (51.2%, 42.4–60.1)
Progressive disease	17 (13.9%, 7.8–20.1)	20 (16.3%, 9.7–22.8)
Nonevaluable	13 (10.7%, 5.2–16.1)	6 (4.9%, 1.1 - 8.7)
Objective response rate	31 (25.4%, 17.7–33.1)	34 (27.6%, 19.7–35.5)
p-value [b]	0.6929	
Disease control rate	92 (75.4%, 67.8 - 83.1)	97 (78.9%, 71.6 - 86.1)
p-value [b]	0.5208	

[a] Data are n (%, 95% CI). [b] P-value is calculated by Exact Cochran-Mantel-Haenszel test.

Table S6. Safety overview (JVDB/JVCZ).

Adverse Event Category	Number (%) of patients<br>JVDB ramucirumab monotherapy<br>Arm 1<br>8 mg/kg<br>Q2W<br>n = 38	 <br>Arm 2<br>12 mg/kg<br>Q2W<br>n = 42	 <br>Arm 3<br>6 mg/kg<br>QW<br>n = 41	 <br>Arm 4<br>8 mg/kg<br>D1D8-Q3W<br>n = 40	 <br>Total<br>N = 161	 JVCZ ramucirumab + paclitaxel 80 mg/m^2<br>Arm 1<br>ramucirumab<br>8 mg/kg +<br>paclitaxel<br>Q2W<br>n = 120	 <br>Arm 2<br>ramuciru<br>mab<br>12 mg/kg +<br>paclitaxel<br>Q2W<br>n = 123	 <br>Total<br>N = 243
**Patients with ≥1 TEAE**	31 (81.6)	32 (76.2)	36 (87.8)	32 (80.0)	131 (81.4)	116 (96.7)	118 (95.9)	234 (96.3)
Related to study treatment [a]	16 (42.1)	14 (33.3)	24 (58.5)	15 (37.5)	69 (42.9)	106 (88.3)	104 (84.6)	210 (86.4)

**Patients with ≥1 Grade ≥3 TEAE**	14 (36.8)	18 (42.9)	18 (43.9)	16 (40.0)	66 (41.0)	80 (66.7)	87 (70.7)	167 (68.7)
Related to study treatment [a]	6 (15.8)	5 (11.9)	8 (19.5)	5 (12.5)	24 (14.9)	63 (52.5)	67 (54.5)	130 (53.5)
**Patients with ≥1 SAE** [b]	10 (26.3)	9 (21.4)	10 (24.4)	14 (35.0)	43 (26.7)	31 (25.8)	47 (38.2)	78 (32.1)
Related to study treatment [a]	3 (7.9)	4 (9.5)	5 (12.2)	5 (12.5)	17 (10.6)	17 (14.2)	23 (18.7)	40 (16.5)
**Patients who discontinued study treatment because of AEs**	4 (10.5)	4 (9.5)	2 (4.9)	6 (15.0)	16 (9.9)	11 (9.2)	23 (18.7)	34 (14.0)
Related to study treatment [a]	1 (2.6)	2 (4.8)	2 (4.9)	3 (7.5)	8 (5.0)	7 (5.8)	13 (10.6)	20 (8.2)
**Patients who discontinued study because of SAEs**	3 (7.9)	3 (7.1)	1 (2.4)	3 (7.5)	10 (6.2)	7 (5.8)	12 (9.8)	19 (7.8)
Related to study treatment [a]	1 (2.6)	2 (4.8)	1 (2.4)	2 (5.0)	6 (3.7)	4 (3.3)	6 (4.9)	10 (4.1)
**Patients who died because of AEs on therapy or within 30 days of treatment discontinuation** [c]	1 (2.6)	1 (2.4)	3 (7.3)	2 (5.0)	7 (4.3)	8 (6.7)	9 (7.3)	17 (7.0)
Related to study treatment [a]	0	0	2 (4.9)	0	2 (1.2)	4 (3.3)	5 (4.1)	9 (3.7)

Abbreviations: AE = adverse event; D1D8 = Day 1 and Day 8 of a 3-week cycle; *N* = number of patients in the safety population; Q2W = every 2 weeks; Q3W = every 3 weeks; QW = weekly; SAE = serious adverse event; TEAE = treatment-emergent adverse event. [a] Relatedness to study treatment was assessed by the investigator. [b] One patient on Arm 4 experienced an SAE prior to the first dose of ramucirumab. [c] Deaths are also included as SAEs and discontinuations due to AEs.

Table S7. Treatment-emergent AESIs for ramucirumab (regardless of causality) reported in the safety population of (A) ramucirumab monotherapy JVDB, and (B) combination therapy JVCZ studies.

A	JVDB—ramucirumab monotherapy[a]									
**AESI category** *MedDRA preferred term*	**Arm 1 8 mg/kg Q2W (*N* = 38)**		**Arm 2 12 mg/kg Q2W (*N* = 42)**		**Arm 3 6 mg/kg QW (*N* = 41)**		**Arm 4 8 mg/kg D1D8-Q3W (*N* = 40)**		**Total (*N* = 161)**	
	**All grades *n* (%)**	**Grade ≥3 *n* (%)**	**All grades *n* (%)**	Grade ≥3 *n* (%)	All grades *n* (%)	Grade ≥3 *n* (%)	All grades *n* (%)	Grade ≥3 *n* (%)	All grades *n* (%)	Grade ≥3 *n* (%)
**Patients with any treatment-emergent AESI**	18 (47.4)	7 (18.4)	16 (38.1)	5 (11.9)	22 (53.7)	11 (26.8)	13 (32.5)	7 (17.5)	69 (42.9)	30 (18.7)
**Bleeding / hemorrhage events** [b]	4 (10.5)	1 (2.6)	8 (19.0)	0 (0.0)	14 (34.1)	6 (14.6)	5 (12.5)	2 (5.0)	31 (19.3)	9 (5.6)
Epistaxis	1 (2.6)	(0.0)	1 (2.4)	0 (0.0)	5 (12.2)	0 (0.0)	2 (5.0)	0 (0.0)	9 (5.6)	0 (0.0)
Gastric hemorrhage	0 (0.0)	0 (0.0)	0 (0.0)	0 (0.0)	3 (7.3)	2 (4.9)	0 (0.0)	0 (0.0)	3 (1.9)	2 (1.2)
**Hypertension**[b]	5 (13.2)	3 (7.9)	2 (4.8)	1 (2.4)	7 (17.1)	2 (4.9)	5 (12.5)	2 (5.0)	19 (11.8)	8 (5)
**Liver injury / failure** [b]	5 (13.2)	1 (2.6)	6 (14.3)	2 (4.8)	2 (4.9)	2 (4.9)	1 (2.5)	1 (2.5)	14 (8.7)	6 (3.7)
Alanine aminotransferase increased	3 (7.9)	1 (2.6)	5 (11.9)	1 (2.4)	0 (0.0)	0 (0.0)	0 (0.0)	0 (0.0)	8 (5.0)	2 (1.2)
Aspartate aminotransferase increased	5 (13.2)	1 (2.6)	5 (11.9)	1 (2.4)	2 (4.9)	1 (2.4)	0 (0.0)	0 (0.0)	12 (7.5)	3 (1.9)
Blood bilirubin increased	3 (7.9)	0 (0.0)	0 (0.0)	0 (0.0)	0 (0.0)	0 (0.0)	0 (0.0)	0 (0.0)	3 (1.9)	0 (0.0)
**Proteinuria**	4 (10.5)	0 (0.0)	1 (2.4)	0 (0.0)	2 (4.9)	1 (2.4)	2 (5.0)	0 (0.0)	9 (5.6)	1 (0.6)
**GI perforation**[b]	2 (5.3)	1 (2.6)	0 (0.0)	0 (0.0)	1 (2.4)	0 (0.0)	1 (2.5)	1 (2.5)	4 (2.5)	2 (1.2)
**Infusion related reaction**	2 (5.3)	1 (2.6)	0 (0.0)	0 (0.0)	2 (4.9)	0 (0.0)	0 (0.0)	0 (0.0)	4 (2.5)	1 (0.6)
**Renal failure**	2 (5.3)	0 (0.0)	1 (2.4)	0 (0.0)	0 (0.0)	0 (0.0)	1 (2.5)	0 (0.0)	4 (2.5)	0 (0.0)

Blood creatinine increased	2 (5.3)	0 (0.0)	1 (2.4)	0 (0.0)	0 (0.0)	0 (0.0)	1 (2.5)	0 (0.0)	4 (2.5)	0 (0.0)

**B**	**JVCZ—combination therapy[c]**					
**AESI category** *MedDRA preferred term*	**Arm 1 ramucirumab 8 mg/kg + paclitaxel 80 mg/m^2 *N* = 120**		**Arm 2 ramucirumab 12 mg/kg + paclitaxel 80 mg/m^2 *N* = 123**		**Total (*N* = 243)**	
	All grades *n* (%)	Grade ≥3 *n* (%)	All grades *n* (%)	Grade ≥3 *n* (%)	All grades *n* (%)	Grade ≥3 *n* (%)
**Patients with any treatment-emergent AESI**	68 (56.7)	20 (16.7)	77 (62.6)	28 (22.8)	145 (59.7)	48 (19.8)
**Bleeding / hemorrhage events[b]**	39 (32.5)	3 (2.5)	37 (30.1)	7 (5.7)	76 (31.3)	10 (4.1)
Epistaxis	28 (23.3)	0 (0.0)	26 (21.1)	0 (0.0)	54 (22.2)	0 (0)
Gastric hemorrhage	2 (1.7)	1 (0.8)	4 (3.3)	3 (2.4)	6 (2.5)	4 (1.6)
Hematemesis	0 (0.0)	0 (0.0)	4 (3.3)	1 (0.8)	4 (1.6)	1 (0.4)
**Hypertension**	21 (17.5)	10 (8.3)	24 (19.5)	10 (8.1)	45 (18.5)	20 (8.2)
**Liver injury / failure[b]**	15 (12.5)	3 (2.5)	29 (23.6)	10 (8.1)	44 (18.1)	13 (5.3)
Alanine aminotransferase increased	7 (5.8)	2 (1.7)	14 (11.4)	1 (0.8)	21 (8.6)	3 (1.2)
Aspartate aminotransferase increased	8 (6.7)	3 (2.5)	21 (17.1)	2 (1.6)	29 (11.9)	5 (2.1)
Blood bilirubin increased	2 (1.7)	0 (0.0)	8 (6.5)	6 (4.9)	10 (4.1)	6 (2.5)
Gamma-glutamyltransferase increased	3 (2.5)	0 (0.0)	4 (3.3)	2 (1.6)	7 (2.9)	2 (0.8)
**Proteinuria**	9 (7.5)	2 (1.7)	9 (7.3)	1 (0.8)	18 (7.4)	3 (1.2)

**Venous thromboembolic events[b]**	7 (5.8)	1 (0.8)	3 (2.4)	1 (0.8)	10 (4.1)	2 (0.8)
**GI perforation[b]**	4 (3.3)	2 (1.7)	0 (0.0)	0 (0.0)	4 (1.6)	2 (0.8)

Abbreviations: AESI = adverse event of special interest; D1D8-Q3W = Day 1 and Day 8 of a 3-week cycle; GI = gastrointestinal; MedDRA = Medical Dictionary for Regulatory Activities; *N* = number of patients in the safety population; Q2W = every 2 weeks; Q3W = every 3 weeks; QW = weekly. [a] For JVDB, AESIs (by MedDRA preferred term) occurring in ≥5% of patients in at least 1 treatment arm of the safety population are reported and ordered by decreasing frequency of AESI in the total column. [b] AESI categories for JVDB and JVCZ are consolidated terms incorporating the multiple MedDRA preferred terms. [c] For JVCZ, AESIs (by MedDRA preferred term) occurring in ≥2% of patients in at least 1 treatment arm of the safety population are reported and ordered by decreasing frequency of AESI in the total column.

(A) JVDB study

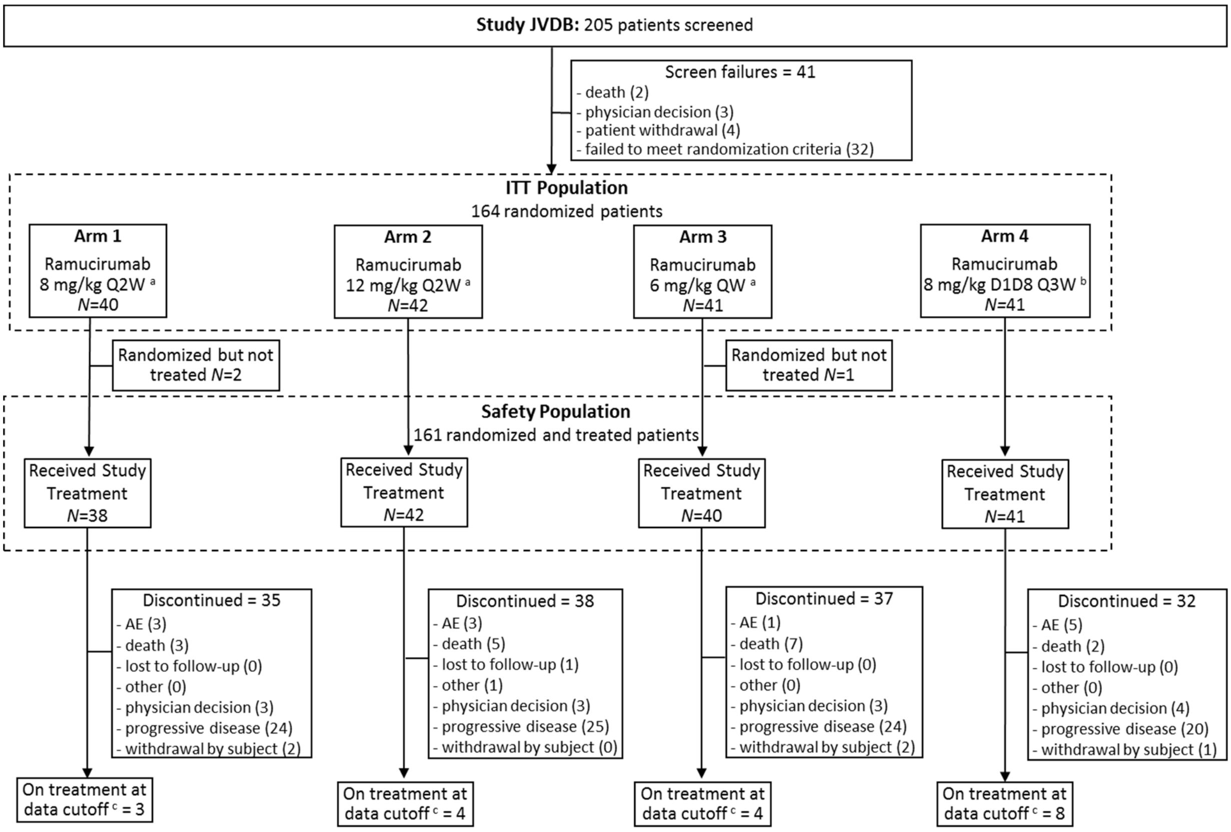

[a] Ramucirumab was administered on a 28-day cycle. [b] Ramucirumab was administered on a 21-day cycle. [c] The data cutoff date was 18 November 2016.

(B) JVCZ study

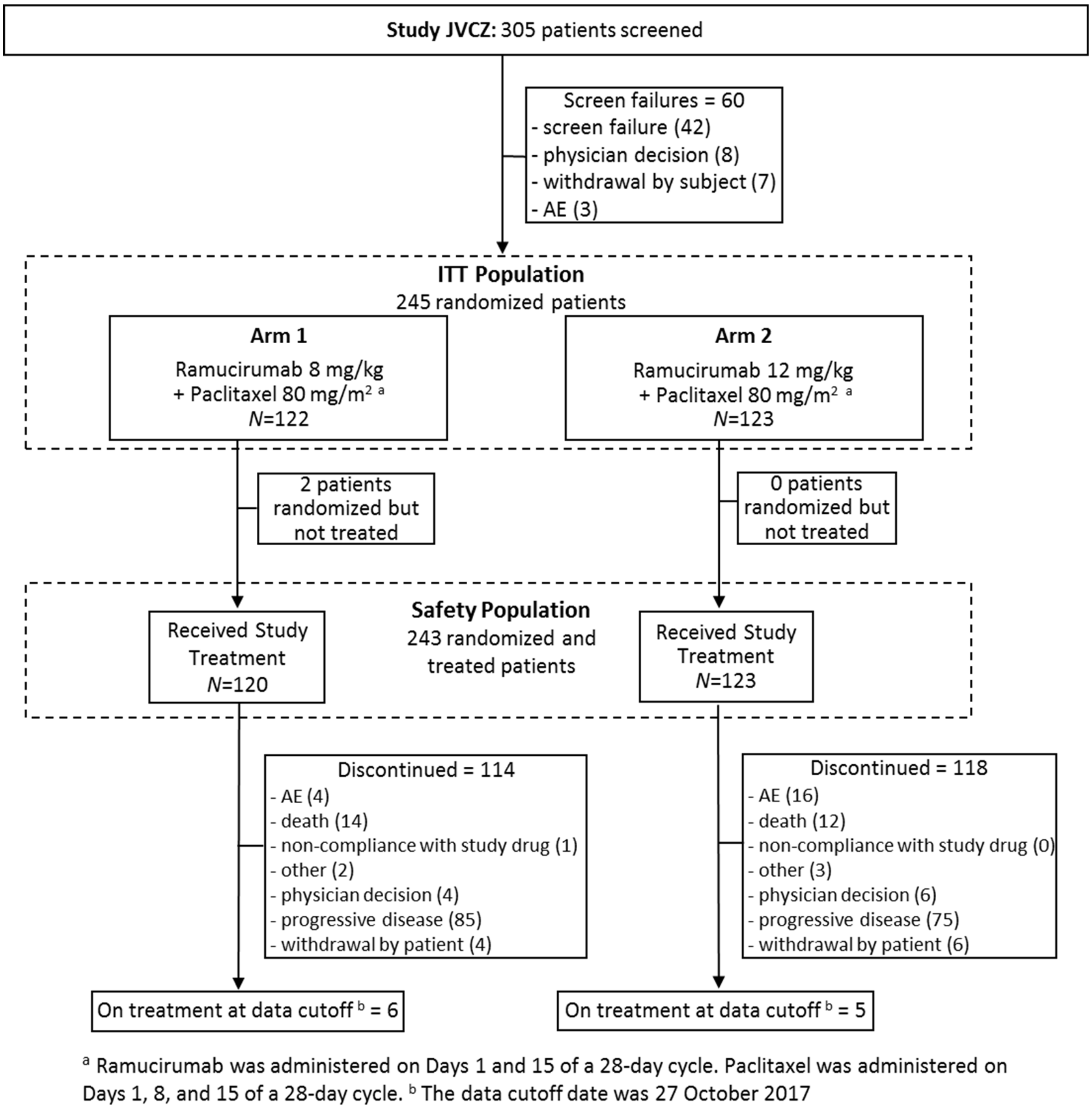

Figure S1. CONSORT diagrams. (**A**) Study I4T-MC-JVDB (**B**) Study I4T-MC-JVCZ. Abbreviations: AE = adverse events; D1D8 Q3W = Day 1 and Day 8 of a 3-week cycle; ITT, intention-to-treat; *N* = number of randomized patients in a given group; Q2W = every 2 weeks; Q3W = every 3 weeks; QW = weekly.
